# Natural Health Products (NHP’s) and Natural Compounds as Therapeutic Agents for the Treatment of Cancer; Mechanisms of Anti-Cancer Activity of Natural Compounds and Overall Trends

**DOI:** 10.3390/ijms21228480

**Published:** 2020-11-11

**Authors:** Benjamin Scaria, Siddhartha Sood, Christopher Raad, Jana Khanafer, Rahul Jayachandiran, Alaina Pupulin, Sahibjot Grewal, Michael Okoko, Mansi Arora, Lauren Miles, Siyaram Pandey

**Affiliations:** Department of Chemistry and Biochemistry, University of Windsor, Windsor, ON N9B 3P4, Canada; scariab@uwindsor.ca (B.S.); soods@uwindsor.ca (S.S.); raadc@uwindsor.ca (C.R.); khanafej@uwindsor.ca (J.K.); jayachar@uwindsor.ca (R.J.); pupulin1@uwindsor.ca (A.P.); grewa127@uwindsor.ca (S.G.); okoko2@uwindsor.ca (M.O.); arora12a@uwindsor.ca (M.A.); milesl@uwindsor.ca (L.M.)

**Keywords:** complementary and alternative medicine (CAM), cancer, natural compound, integrative oncology, natural health product (NHP), apoptosis, drug–drug interaction, neoplasm, anti-cancer effect

## Abstract

Most cancer therapeutics, such as tubulin-targeting chemotherapy drugs, cause cytotoxic, non-selective effects. These harmful side-effects drastically reduce the cancer patient’s quality of life. Recently, researchers have focused their efforts on studying natural health products (NHP’s) which have demonstrated the ability to selectively target cancer cells in cellular and animal models. However, the major hurdle of clinical validation remains. NHP’s warrant further clinical investigation as a therapeutic option since they exhibit low toxicity, while retaining a selective effect. Additionally, they can sensitize cancerous cells to chemotherapy, which enhances the efficacy of chemotherapeutic drugs, indicating that they can be utilized as supplemental therapy. An additional area for further research is the investigation of drug–drug interactions between NHP’s and chemotherapeutics. The objectives of this review are to report the most recent results from the field of anticancer NHP research, and to highlight the most recent advancements in possible supplemental therapeutic options.

## 1. Introduction

Cancer is amongst the most prevalent diseases in modern-day society. Nearly 10 million people died from the disease in 2017, and every sixth death in the world is attributed to cancer [1]. Following a cancer diagnosis, healthcare professionals will decide on the best course of treatment, often combining multiple types of treatments or therapies. Removal of the tumour is often done via surgery and referred to as the primary treatment, followed by adjuvant therapy such as chemotherapy to lower the chances of tumour formation in the future. Neoadjuvant therapy refers to treatment given before the primary treatment and is also used to lower the chances of a future recurrence of a malignant tumour [2]. Examples of adjuvant therapies include chemotherapy, hormone therapy, radiation therapy, and immunotherapy amongst others [2]. Chemotherapy in particular is a widely used adjuvant treatment for a wide variety of cancers. However, there are significant and long-term toxic side-effects of chemotherapies that have been reported in the literature. For example, in breast cancer, the use of chemotherapy is still prevalent as a form of adjuvant therapy for the treatment of the disease. However, there are several notable side effects of chemotherapy use in breast cancer patients; this is a significant issue because there are several million breast cancer survivors in the United States alone, and the risk of disease recurrence is high [3]. Side-effects from adjuvant chemotherapy can be short-term which refers to side-effects that occur during a course of treatment and last temporarily or do no last beyond the period of treatment, or they can be long-term effects which typically are experienced after cessation of treatment and lasts for a significant period of time or permanently [4]. Significant side-effects of chemotherapy use include ovarian failure in premenopausal women, weight gain, decrease in bone density that increases risk of fractures, sensory and motor neuropathy leading to pain and paresthesia, and left ventricular dysfunction [3,4]. Neurocognitive dysfunction (“chemo-brain”) is estimated to be reported by 75% of women who receive chemotherapy within 2 years after treatment and is reported as a decline in attention, memory, and concentration [3,4]. Another severe yet rare long-term side effect of chemotherapy treatment is the development of a secondary hematological malignancy, such as leukemia [3,4]. Radiation therapy also has certain side-effects, such as neurological deficits due to vascular damage and fibrosis of neural structures depending on the total dose and other factors [5]. Other chemotherapeutic agents that can penetrate the blood–brain barrier, such as cytarabine, methotrexate, nitrosoureas, and procarbazine can induce acute encephalopathy. Radiation to the brain and neck along with chemotherapeutic agents such as cyclosporine, doxorubicin, methotrexate, and cisplatin (when administered along with other chemotherapeutic agents) can increase the risk of stroke [5]. All of the above are side-effects of conventional therapies used in the treatment of a broad spectrum of cancer diseases. It is self-evident and understandable through the descriptions of these unwanted yet unavoidable side-effects that they can significantly hamper daily routines and lower the quality of life of patients over both the short and long-term. Certain natural health products (NHP’s) might offer a solution that alleviates the side-effects, at least partially, without interacting negatively with the conventional adjuvant therapies being administered.

**Figure 1:**
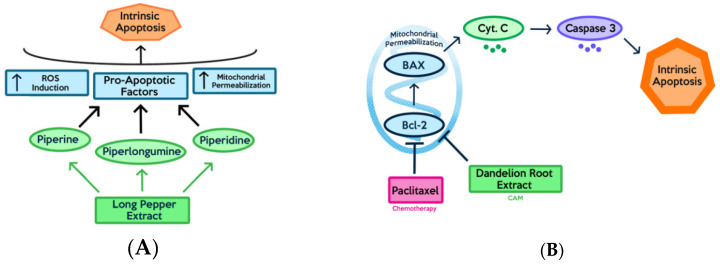
Figure depicts anticancer effects and specific mechanisms of action of conventional therapy and compounds found in natural extracts: (**A**) Long pepper extract is able to induce apoptosis via multiple pathways through the activity of multiple compounds contained within the extract; (**B**) Combination treatment of paclitaxel and dandelion root extract (DRE) can suppress the activity of the anti-apoptotic protein Bcl-2, eventually inducing apoptosis.

When patients opt to use a non-mainstream treatment as a supplementary or additional treatment modality in addition to the conventional adjuvant therapy such as chemotherapy (CT) and radiation therapy (RT), it is referred to as complementary medicine. Alternative medicine is the term employed when the non-mainstream treatment replaces the conventional modes of treatment completely, such as a patient opting to forego chemotherapy for homeopathic treatments. Integrative medicine combines complementary and conventional approaches to promote holistic health that emphasizes “mental, emotional, functional, spiritual, social and community aspects” of health [6]. Although there are many claims with regard to the efficacy of complementary and alternative treatments, it is important to examine claims made by practitioners and proponents in light of the scientific evidence. According to the national centre for complementary and integrative medicine (NCCIH), NHP’s include products such as herbs (botanicals), vitamins and minerals, and probiotics. According to a 2012 National Health Interview Survey (NHIS), 17.7% of American adults used NHP’s, which represented the most popular option within the various complementary treatments that are available [6]. Patients who use CAM are most likely doing it to actively cope with their disease and make conscious decisions towards health and general well-being by making changes to their lifestyle and increasing social support. Individuals who use active coping strategies reported having greater emotional and physical well-being [7,8,9]. One of the primary concerns surrounding the use of CAM therapies is the potential for negative interactions with conventional treatments, which can lower the efficacy of treatment. The National Cancer Institute resounds this warning as a recommendation, “Cancer patients who are using or considering using complementary or alternative therapy should talk with their doctor or nurse. Some therapies may interfere with standard treatment or even be harmful. It is also a good idea to learn whether the therapy has been proven to do what it claims to do [10].” In the case of cancer, an example of negative interaction between a CAM therapy and conventional treatment would be that of St. John’s wort. St. John’s wort is one of the most commonly used herbal medicines. It is often used to treat depression and anxiety; however, it can decrease the efficacy of certain anticancer drugs and antineoplastic drugs as a result of hyperforin (active compound in St. John’s wort) inducing the CYP3A4 enzyme system and P-glycoprotein drug transporter which subsequently increases hepatic metabolism and leads to increased clearance of the drugs [10,11]. On the other end of the spectrum, CAM therapies can have positive synergistic interactions with convention treatment modalities (Figure 1). An example of this would be that of milk thistle and chemotherapy drugs. An active compound found within milk thistle, which is often used by patients with liver disease, is silymarin. It is reported that this active compound has a hepatoprotective effect used as an adjunct with cancer therapies, and furthermore that it exhibits synergistic effects when used in combination with doxorubicin, cisplatin, and carboplatin [12]. Therefore, it is important for healthcare providers and patients alike to be aware of the risks and benefits conferred through the use of NHP’s.

The prevalence of complementary and alternative medicine (CAM) use is an important factor that warrants attention. A study conducted at the North Carolina Cancer Hospital (NCCH) found that 85% of survey respondents indicated that they used CAM treatment following a cancer diagnosis, in the periods during and after the initial treatment [13]. The prevalence of CAM use amongst cancer patients is estimated to between 60–80% depending on factors such as methods of sampling used, and the time period of the study [13]. Although the prevalence rate of CAM use amongst cancer patients is high, the nondisclosure rate, which measures what percent of cancer patients do not report their use of CAM treatments to their healthcare providers remains high at an estimated 80% [13]. This is a significant cause for concern in cancer treatment due to the reality of the potential for negative antagonistic interactions with conventional cancer treatments, especially in herbal treatments and plant-based extracts used as complementary supplemental therapy [14,15]. However, the evaluation of the nature of drug–drug interactions between NHP’s and conventional cancer therapy is underreported in the literature. As a result, there exists both a need and various potential benefits to a summary of the current literature and the identification of this knowledge gap in order to guide future research.

In the following sections, the following topics are discussed: challenges with conventional therapeutics, advancements in anticancer NHP’s, and a breakdown of compounds contained within those NHP’s.

## 2. Natural Health Products and Natural Compounds Used in the Treatment of Breast Cancer

### 2.1. Prevalence Statistics, Prognosis, and Downsides of Conventional Treatments

Among women, breast cancer is the most commonly diagnosed type of cancer and accounts for the highest cancer mortality rate globally [16]. Breast cancer can be caused by genetic factors; however, environmental factors such as alcohol consumption, obesity and lack of physical activity can play a significant role in the establishment and progression of the disease. Thus, it is necessary for physicians to employ a multidisciplinary approach to combating cancer that focuses on disease prevention, early detection, quick intervention, and continued observation throughout treatment. Aside from primary intervention through alterations in diet and lifestyle, secondary prevention techniques such as mammography, ultrasonography, magnetic resonance imaging, and breast self-examination all assist with early detection of tumours [17].

While there are many types of breast cancer, two common and well-studied classes include hormone receptor-positive and triple-negative breast cancers. Hormone receptor-positive breast cancer cells contain receptors that respond to estrogen and/or progesterone hormones. Thus, treatment for these types of cancer involve molecularly targeting and blocking hormone-receptor pathways through hormone therapy with adjuvant therapies including ovarian suppression, selective estrogen receptor modulators (SERMs) and aromatase inhibitors [18,19]. On the other hand, triple-negative breast cancer cells lack these hormone receptors and thus, do not respond to hormone therapy. Because they lack certain drug targets, triple-negative cancers are often associated with an unfavourable prognosis as chemotherapy serves as the only valid systemic treatment option [20]. In addition to the breast cancer type, treatment can also vary depending on which stage the cancer is progressing through. Early-stage breast cancer is typically targeted using breast-conserving surgery and radiation therapy, which can decrease local tumour recurrence. However, if the cancer reaches a locally advanced stage, physicians utilize a combination of techniques including chemotherapy, followed by breast-conserving surgery and radiation therapy. If the cancer progresses to a metastatic stage, chemotherapy is often administered to induce a prompt reaction. For late-stage metastatic cancer, treatment options must consider both quality and length of life depending on the prognosis [21]. 

Although these options serve as effective treatments, they lack specificity for their targets and can often result in systemic toxicity. Inadequate drug doses in target areas and can even lead to the generation of drug-resistant tumour cells. This can cause several adverse side-effects and, therefore, it is essential that more specific therapies are researched and developed, allowing the targeting of features specific to cancer cells [22]. Recently, researchers have investigated the possibility of targeting cellular vulnerabilities specific to cancer as a new treatment option. As cancer cells require high-energy production to rapidly proliferate, they can become susceptible to oxidative stress and mitochondrial membrane destabilization [23]. A therapeutic agent that is able to target the vulnerabilities of a cancer cell could induce a controlled and specific physiological process called apoptosis, which ultimately leads to the death of the cancer cell [24].

### 2.2. Bioactive Compounds

**Figure 2:**
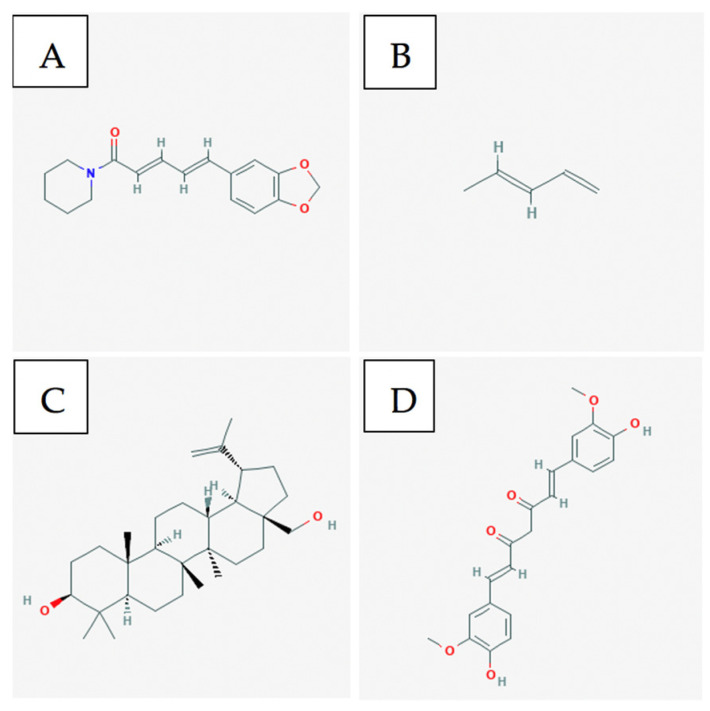
A few of the bioactive compounds with known anti-cancer activity towards breast cancer: (**A**) Piperine; (**B**) Piperylene; (**C**) Betulin; (**D**) curcumin [28,29,30,31].

Piperine and piperylene (Figure 2A and B respectively) are both major constituents of Piper nigrum plant. On its own, this component has been proven to be efficacious against ER+ and triple-negative breast cancer cell lines in-vitro and in xenograft models [25]. Greenshields et al. have shown that piperine inhibited proliferation and induced apoptosis by collapsing the mitochondrial membrane potential. They have further shown that the compound inhibited tumour growth without any observed adverse effects in nude mice. Piperine induced apoptosis independently of caspases and oxidative stress [26]. Piperine has been shown to act as a bioenhancer as well as to increase the bioavailability of chemotherapeutics by augmenting drug absorption and decreasing drug metabolism through the inhibition of cytochrome P-450/CYP3A4. When piperine was used against triple-negative breast cancer cells (MDA-MB-231) in combination with Paclitaxel, a chemotherapeutic, the therapeutic agents were able to act in synergy to enhance cytotoxicity against cancer cells. Piperine, in combination with Paclitaxel, showed a significantly lower IC50 as compared to just Paclitaxel individually. This demonstrates Piperine’s ability to enhance the anti-cancer effect of chemotherapy. By improving the bioavailability of chemotherapeutic drugs, piperine reduces the required drug dose and consequently, the associated side effects of the drug [27].

The leaves of the native Asian flower Hibiscus possess anti-cancer components, such as flavonoids, triterpenoids, anthocyanin, and phenolic compounds [32,33,34,35]. Particularly, Hsu et al. have shown that the triterpenoids betulin (Figure 2C), its derivatives, and betulinic acid selectively induced cell death and reduced cell migration in ER+ and TN breast cancer models. The authors have found that betulin induced apoptosis via a p21-related pathway. Furthermore, betulin and betulinic acid enhanced the production of a TAp63 in TNBC, a regulatory protein involved in tumour suppression. Consequently, pro-apoptotic proteins such as BAX were produced, resulting in cell death [36].

After characterizing the constituents of the leaves and seeds of the Annona muricata plant, researchers found that acetogenin, a major anti-cancer component, was present. This pure compound is capable of preventing cancerous cells’ access to ATP [37]. The secondary metabolite disrupts cellular respiration by inhibiting complex 1 in the electron transport chain, ultimately resulting in selective apoptosis [38].

Curcumin (Figure 2D) is a major constituent of turmeric and has been shown to possess anti-cancer effects against multiple cancer cell lines [39]. Pignanelli et al. have shown that by inducing oxidative stress, two analogs of curcumin were capable of selectively inducing apoptosis in MDA-MB-231, MDA-MB-468, and SUM149, three triple-negative breast cancer cell lines, in-vitro. It was observed that in breast cancer, these analogs induced apoptosis more effectively than taxol. Furthermore, an MDA-MB-231 study on immunocompromised mice revealed that curcumin analogs are well-tolerated and effectively inhibit tumour growth [40].

In addition to the above mentioned compounds, there have been interesting studies with various ginsenosides that have shown anti-cancer activities on breast cancer. Ginsenosides, a class of natural triterpenoid saponins, come from the Panax ginseng Meyer (ginseng) plant. Ginsenoside Rg3 is a member of the ginsenoside family with two distinct epimers: 20(S)-ginsenoside Rg3 (SRg3) and 20(R)-ginsenoside Rg3 (RRg3). Rg3 has been shown to induce apoptosis and induce GO/G1 cell cycle arrest in breast cancer cells; however, the specific effects of each individual epimer of Rg3 had not been characterized in breast cancer. Nakhjavani et al. investigated the stereoselective effects of the two epimers of Rg3 and found that inhibition of proliferation is dependent upon stereoselectivity in triple negative MDA-MB-231 breast cancer cells. The epimer with the noted effect, SRg3, might be inhibiting proliferation by inhibiting Aquaporin 1 (AQP1) water flux by binding to it. Furthermore, it was determined that SRg3 induced GO/G1 cell cycle arrest. Inhibition of AQP1 inhibits the G1-S transition, which then has the effect of arresting the cells in the GO/G1 phase, which leads to the inhibition of proliferation [41]. Rg3 was also able to activate Caspase-3 and degrade PARP (downstream target of caspases) in MDA-MB-231 cells through the generation of reactive oxygen species (ROS). Rg3 was also able to increase the ratio of pro-apoptotic protein BAX to the anti-apoptotic protein Bcl-2, inhibit binding of NF-kB to DNA, and prevented the phosphorylation of Akt and ERK, which are upstream activators of NF-kB. All these effects were observed in a study involving Rg3 and MDA-MB-231 breast cancer cells. Rg3 was able to minimize the oncogenic effect of mutant P53, lower the expression of Bcl-2, which led to induction of apoptosis. Furthermore, Rg3 has been able to inhibit angiogenesis and tumour growth in breast cancer cells [42]. Ginsenoside F2 is a metabolite of ginsenoside Rb1. Rb1 is converted to F2 after oral ingestion. Mai et al. determined that F2 was able to inhibit proliferation of breast cancer stem cells (CSC’s) via apoptosis. F2 induced protective autophagy and apoptotic cell death in breast CSC’s via the upregulation of P53 [43]. Ginsenoside Rp1 is reported to have the most potent chemopreventive and antimetastatic effect of all ginsenosides. Furthermore, it has been reported that it induces apoptosis via the activation of caspases-3, -8, and -9, and induce cell cycle arrest in HeLa cells. Additionally, it has also been shown to inhibit the NF-kB pathway, and it has been suspected to inhibit the IGF-1R/Akt pathway. IGF-1r is known to play a major role in cancer progression, proliferation, and survival. Kang et al. reported that treatment of breast cancer cells with ginsenoside Rp1 inhibited cancer cell growth proliferation, colony formation, and induced cell cycle arrest. Treatment with Rp1 resulted in the degradation of/loss in stability of IGF-1R and reduced expression of pAkt [44]. Furthermore, an interesting study was published with anti-cancer activity of bacopasides I and II in breast cancer. The results indicate that bacopasides I and II synergistically reduced the proliferation and viability in four different breast cancer cell lines [45].

## 3. Natural Health Products and Natural Compounds Used in the Treatment of Melanoma

### 3.1. Prevalence Statistics, Prognosis, and Downsides of Conventional Treatments

Global incidence of Melanoma has greatly increased in the last few decades. In 2020, an estimated 8000 Canadians will be diagnosed with melanoma skin cancer, with 1300 Canadians facing mortality as a result [46]. Of all skin cancers, melanoma is the most likely to become metastatic, which is why it has a poor prognosis [47]. The 5-year overall survival rate for patients with metastatic melanoma is typically between 5–19% [47]. Surgical resection is typically used in early melanoma; however, this is not possible in advanced melanoma [48]. In late-stage cases, radiotherapy and systemic treatments such as chemotherapy and immunotherapies are used. Currently, dacarbazine is the only approved single-agent chemotherapy for melanoma. However, it has shown poor responses rates [49]. Immunotherapies and targeted therapies have emerged in the last decade, including ipilimumab and vemurafenib [50]. Natural Health Products (NHPs) for use in melanoma has been researched due to minimal toxicity and long-term potential [51]. NHPs such as dandelion root extract [52] have shown anti-cancer cytotoxicity. Purified compounds such as curcumin [53] and curcumin analogs [39], and vincristine and vinblastine [54] have also shown anti-cancer effects. In order to search for effective complementary treatments for this aggressive cancer, many NHPs and purified compounds are currently being tested in combination with chemotherapeutic drugs in both the pre-clinical and clinical phase. Thus, in this section, we list recent advancements into this area of research.

If melanoma is detected at an early stage, it can be treated by surgical procedures that remove the primary lesions. However, this form of treatment is used far less frequently for advanced stages of melanoma cancer where the melanocytes have become metastatic and spread throughout several parts of the body [48,55]. This problem is further exacerbated due to the difficulty in the diagnosis of this condition as primary melanoma lesions often remain invisible and asymptomatic [56]. Furthermore, metastatic melanoma has a poor prognosis due to the lack of viable therapeutic options as there has been limited success in fighting this cancer using conventional chemotherapies and radiotherapies. Dacarbazine, as previously mentioned, has typically shown poor response rates of between 10% to 20% [49]. Difficulties in treatment with dacarbazine may be explained by the activation of signal specific pathways to escape the drug’s mechanism of action [57]. The resistance to chemotherapy that melanoma cancer cells show is a multifactorial process as these cells disrupt their drug transport pathway systems, downregulate apoptotic pathways and alter enzymes involved with their metabolism to prevent the drug from producing its cytotoxic effects [58]. Moreover, chemotherapy in melanoma has often shown side effects such as thrombocytopenia and neutropenia [49]. Radiation therapy has also been ineffective in treating melanoma as these tumours are typically radioresistant, therefore weaker hypo-fractionated doses of radiation are administered instead to reduce toxicity [59]. Immunotherapy has been a promising development with cytokines and immune checkpoint inhibitors having shown success in reducing the ability for metastatic melanoma to avoid the immune response [60]. However, adverse effects relating to overactivation off the immune system such as dermatitis and colitis have been observed with their use [61]. Thus, due to the issues relating to resistance and toxicity with various current treatments, NHPs may serve as a beneficial complementary treatment due to their potential to enhance efficacy and protect healthy cells [62].

### 3.2. Anticancer Effects of Natural Compounds In-Vitro and In-Vivo

Found within turmeric, curcumin is a purified compound derived from the Curcuma longa plant which has demonstrated anti-cancer effects [39]. Curcumin has been shown to selectively target melanoma cancer cells by altering the activity of signalling molecules and inducing inflammation pathways. Unfortunately, when tested using in-vivo mouse models, it showed poor bioavailability [39]. Chemical analogues of curcumin such as Compound A were developed as they were more stable and efficacious while showing anti-cancer effects against human melanoma cells either alone or combined with contemporary chemotherapeutics such as paclitaxel [39].

Found within plants from the Berberis genus, berberine is an isoquinoline alkaloid that was tested as a complementary medicine along with doxorubicin, a common chemotherapeutic [51,63]. Results indicated that this combination treatment showed anti-melanoma activity in both in vitro tests and in vivo models of xenografted mice when using the B16F10 melanoma cell line [51,64].

Vinblastine and vincristine are yet another example of purified natural products that have been used against melanoma cancer cells after being derived from the Catharanthus roseus plant [65]. Vinblastine has been tested in combination trials with chemotherapeutics such as cisplatin and dacarbazine and their combined effect is more efficacious to melanoma cells than the effect of any of these single agents alone [66]. Vincristine shows a synergistic effect when it is used in a combination trial with betulinic acid (a pentacyclic triterpene) in both in vitro tests and in vivo models of mice with the B16F10 melanoma cell line [67].

### 3.3. Bioactive Compounds

**Figure 3:**
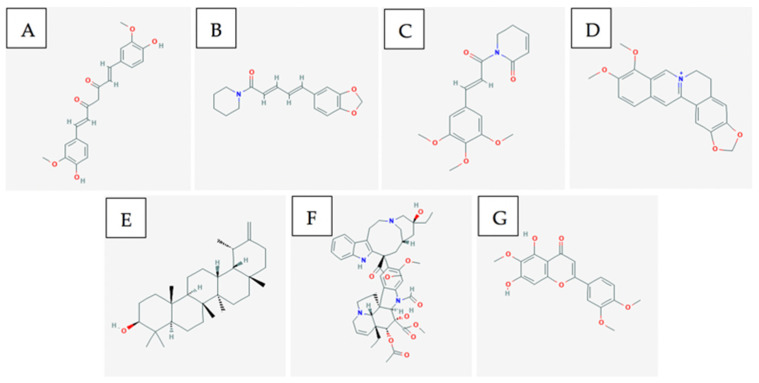
A few of the bioactive compounds with known anti-cancer activity towards melanoma: (**A**) Curcumin; (**B**) Piperine; (**C**) Piperlongumine; (**D**) Berberine; (**E**) Taraxasterol; (**F**) Vincristine; (**G**) Eupatilin. [28,31,69,70,71,72,73].

Major components of Curcuma longa (turmeric) are curcumin, alkaloids, sesquiterpenes, and other phenolic compounds [68]. Curcumin (Figure 3A) is the major component of turmeric that is believed to provide anti-cancer and anti-inflammatory activity [53]. However, some studies have shown that compounds such as turmerines, turmerones, and elemenes have similar activity aside from curcumin [68].

Major components of Piper longum (long pepper) include alkaloids such as: piperine (Figure 3B), piperlongumine, and piperidine [74]. Piperine and piperlongumine have been shown to have significant anti-cancer and anti-oxidative capacity [74]. Specifically, piperine has been shown to upregulate pro-apoptotic markers such as BCL2 and BAX [75]. As well, it has been shown to decrease cytokine levels in-vivo. This ultimately produces both an anti-metastatic and anti-angiogenic effect against melanoma cancer cells which depend on these cytokines for tumour progression [76]. Likewise, it has been shown via xenograft models that piperlongumine (Figure 3C) inhibited proliferation of melanoma, upregulated pro-apoptotic genes such as CDK1NA, and reduced expression of VEGF leading to anti-angiogenic properties [77].

Coptidis rhizoma contains alkaloids, such as berberine, palmatine, coptisine, epiberberine, jatrorrhizine, columamine [78]. Berberine is found in many other Berberis genus plants. Berberine increases AMP-activated protein kinase (AMPK) phosphorylation levels by increasing the production of reactive oxygen species [79]. The activation of these AMPK’s results in decreased extracellular signal-related kinase (ERK) activity and decreased levels of the cyclooxygenase-2 (COX-2) protein [79]. Both proteins are critical for melanoma cancer cell invasion, so by decreasing their signalling activity and levels, berberine (Figure 3D) inhibits the metastatic potential [79].

Major components of dandelion root include taraxasterol (Figure 3E), lupeol, sesquiterpene lactones, and phenolic acids. These chemical compounds can lead to anti-oxidative and anti-inflammatory effects [80] which assist in producing anti-cancer activity.

Catharanthus roseus has been used traditionally in Vietnam as an anti-cancer agent [81]. It contains alkaloids such as vincristine (Figure 3F) and vinblastine that have shown anti-cancer activity. Both compounds are microtubule destabilizing compounds which prevent mitosis from completing [82]. They are a part of a large group of several other vinca alkaloids and they are often used in combination treatments with more common anti-cancer agents.

Ganoderma lucidum is a medicinal mushroom that contains a terpene that exerts apoptotic activity via the induction of oxidative stress in-vitro and has shown a translation of effects in-vivo by a reduction in tumour volume in mouse models [83]. Molecular studies have shown this compound upregulates apoptosis-promoting gene p53 and downregulates oncogene Bcl2 [83].

Plants classified under the Artemisia genus contains the flavonoid Eupatilin (Figure 3G) that has shown dose-dependent apoptotic activity in-vitro using A375 cells [84]. The mechanism of action for this compound is suggested to be the induction of apoptosis via DNA damage and the inhibition of the G2/M cell cycle checkpoint [84]. Molecular studies have suggested that eupatilin upregulates proapoptotic gene Bax while downregulating anti-apoptotic gene Bcl2 [84].

### 3.4. Single vs. Multiple Compounds and Collective Activities Targeting Multiple Pathways

Dandelion root plant material can be resolved into various purified components that show modest anti-cancer activity. In particular, taraxasterol and lupeol are two such compounds as lupeol was shown to reduce mouse melanoma differentiation [85] and taraxasterol was shown to reduce Eppstein–Barr virus early antigen (EBV-EA) [86]. There exists the potential for synergism within dandelion root with not only the main bioactive components taraxasterol and lupeol, but with phenolic acids and sesquiterpenes that are also present.

Coptidis rhizome contains Berberine and Jatrorrhizine, which are two natural compounds that have shown anti-melanoma effects [78]. Berberine shows anti-melanoma activity by preventing the migration of melanoma cells and thereby reducing its metastatic potential [79]. Berberine increases AMP-activated protein kinase (AMPK) phosphorylation levels by increasing the production of reactive oxygen species [79]. The activation of these AMPK’s results in decreased extracellular signal-related kinase (ERK) activity and decreased levels of the cyclooxygenase-2 (COX-2) protein [79]. Both proteins are critical for melanoma cancer cell invasion, so by decreasing their signalling activity and levels, berberine inhibits the melanoma cancer’s metastatic potential. While Berberine is the main compound, jatrorrhizine hydrochloride (JH) has been shown to inhibit the proliferation and neovascularization of C8161 human metastatic melanoma cells. JH suppressed C8161 cell proliferation in a dose-dependent manner, with a half-maximal inhibitory concentration of 47.4 ± 1.6 μmol/L; however, it did not induce significant cellular apoptosis at doses up to 320 μmol/L. Mechanistic studies showed that JH-induced C8161 cell cycle arrest at the G0/G1 transition, which was accompanied by overexpression of the cell cycle-suppressive genes p21 and p27 at higher doses. Moreover, JH reduced C8161 cell-mediated neovascularization in vitro and in vivo and impeded the expression of the gene for VE-cadherin, a key protein in tumour vasculogenic mimicry and angiogenesis [87]. The differential actions of BBR and JH suggest that the mechanism of action of Coptidis rhizome is complex and that these compounds may have a synergistic effect in treating melanoma when combined.

The Piper longum plant is composed of piperine, lignans and volatile oils [63]. Piperine as discussed earlier, is the main anti-cancer agent within this extract, but lignans have previously been shown to be anti-cancerous agents due to their antioxidative, antiproliferative and anti-aromatase effects [88]. As a result, there may be a synergistic anti-melanoma effect due to the combination of these anti-cancer compounds working together. One of the compounds known to contribute to this effect is piperlongumine; within mouse xenograft models, it was shown to reduce expression of VEGF while also upregulating pro-apoptotic genes such as CDK1NA [77].

As mentioned previously, curcumin is the major component of turmeric, and it can selectively target melanoma cells to undergo apoptosis by downregulating IFN-γ induced and TNF-α induced epithelial–mesenchymal transitions (EMT) and inhibiting anti-apoptotic factors such as nuclear factor-κB (NF-κB) plus its downstream factors such as COX-2 and cyclin D1 [89]. It slows down cell proliferation by inhibiting PDE1 and PDE4 while regulating cyclin A, p21, p27 and DNA methyl transferase (DNMT1) levels [89]. In addition, curcumin has an anti-metastatic effect due to its inhibitory effect on focal adhesion kinase (FAK) and matrix-metalloproteinase-2 [89]. It does have poor bioavailability, but this can be improved by increasing its water solubility through the use of liposomes or micelles [89]. Β-elemene, one of the forms of elemene found in turmeric has been shown to have an inhibitive effect on B16F10 murine melanoma cells through cell proliferation assays in vitro, angiogenesis assays in vivo, as well as melanoma growth and metastasis assay in C57BL/6 mice. Vascular endothelial growth factor (VEGF) and CD34 expression were inhibited, preventing proliferation, angiogenesis, and metastasis of melanoma [90]. Aromatic (ar)-turmerone was found to have an anti-melanogenic effect on alpha-melanocyte stimulating hormone (α-MSH) and 3-isobuty-1-methxlzanthine (IBMX)-induced tyrosinase in B16F10 melanoma cells. This effect was found to be greater than that of curcumin, so it is proposed as a treatment for hyperpigmentation [91].

Pancratistatin (PST). PST is a compound extracted from Hymenocallis littoralis from the Amaryllidaceae plant family. In melanoma cells, it has been shown to induce apoptosis by depolarization of the mitochondrial membrane observed through JC-1 staining. PST acts synergistically with tamoxifen (TAM), an estrogen receptor antagonist, to induce apoptosis. This was shown by the increase in reactive oxygen species (ROS) generation in mitochondria of the cancer cells while not affecting healthy cells. The low cytotoxicity and high selectiveness of PST indicates possibility of use in treatment of melanoma [52].

Vincristine and Vinblastine. Both of these natural products are derived from the Catharanthus roseus as previously described. Vincristine has been shown to alter the lysosomal membrane potential in cells by making it more sensitive. Therefore, it is very effective when given in combination with lysosomal destabilizing drugs [82]. Similarly, Vinblastine causes apoptosis in melanoma cells by destabilizing the mitochondrial membrane which causes the release of cytochrome c, a pro-apoptotic factor that activates caspases 3 and 8 [92]. Vinblastine also has a non-mitochondrial dependent intrinsic apoptotic mechanism where it causes an increase of reactive oxygen species (ROS), the release of intracellular Ca^2+^, and the activation of many apoptotic signalling proteins [92]. Both of these apoptotic pathways induced by vinblastine are modulated by the Ras homologous A protein (Rho A) [92].

### 3.5. Combination/Supplemental Therapy Involving Natural Compounds and Chemotherapeutic Drugs

Preclinical combinatory work of NHP and standard chemotherapeutics on melanoma has demonstrated positive results. Compound A, a curcumin analog, has shown it may have a small additive effect and no negative interaction with chemotherapeutics paclitaxel and cisplatin in-vitro [39]. In another study, a similar curcumin analog DM-1 observed promising in-vivo tumour reductions via intrinsic apoptosis in both monotherapy and in combination with dacarbazine [93].

PST has shown dose-dependent induction of apoptosis via mitochondrial depolarization, an effect enhanced by sensitization of cells by tamoxifen [52].

With dacarbazine, a number of natural products have been used both in-vitro and in-vivo. In one study, vitamin D and its analogs were used to modulate the anticancer activity of dacarbazine and cisplatin on A375 human melanoma cells. Specifically, the IC50 of both dacarbazine and cisplatin were reduced with the addition of vitamin D and its analogs, with additional increases in ROS and cell cycle inhibition [94].

The compound saponin purified from sea cucumber has shown to potentiate intrinsic apoptosis in the B16F10 murine melanoma cell line with an enhancement seen when added to dacarbazine regiments [95].

## 4. Natural Health Products and Natural Compounds Used in the Treatment of Leukemia and Lymphoma

### 4.1. Prevalence Statistics, Prognosis, and Downsides of Conventional Treatments

Leukemia is a cancer of the bone marrow and blood forming tissues. There are four main subtypes which include: acute lymphoblastic leukemia, acute myeloid leukemia, chronic lymphocytic leukemia and chronic myeloid leukemia. With grouping the various forms, the cancer has shown to be quite prevalent in society. In Canada, leukemia is diagnosed at a rate of 15 cases per 100,000 people [96]. Approximately 22,510 Canadians are living with or are in remission from leukemia: 13,040 males and 11,470 females [97]. Approximately one in 53 men and one in 72 women are expected to develop some form of leukemia in their lifetime. The five-year age standardized survival rate for leukemia in Canada is 58% for males and 59% in females [97]. This is found to be quite low in comparison to other cancers. In regard to children aged 0 to 14 years, leukemia is the most prevalent cancer [98]. The overall success in management despite some improvements over the years is still uninspiring. It is estimated that in 2020, 6900 Canadians will be diagnosed with leukemia and 3000 Canadians will die from it. Advancements must be made to improve the overall prognosis.

Lymphoma refers to cancer of the lymphocytes, which are a type of white blood cell found in the lymphatic system. Lymphoma is found in children and adults with two main types: Hodgkin’s lymphoma and Non-Hodgkin’s lymphoma (NHL). It is estimated that 43,335 Canadians are either living with, or in their remission from lymphoma. Of these, 36,175 are non-Hodgkin’s lymphoma and 7160 are Hodgkin’s lymphoma [97]. The average age of diagnosis is 39 and 66 years for Hodgkin’s and non-Hodgkin’s lymphoma, respectively. Non-Hodgkin’s lymphoma is the sixth most commonly diagnosed cancer in Canada. The probability of one developing Hodgkin’s lymphoma is only one in 432 in males and one in 498 in females. With regard to non-Hodgkin’s, these chances increase significantly with a probability of one in 43 for men and one in 50 for women in their lifetime. There has been noticeable success in the management of Hodgkin’s lymphoma with the five-year standard survival rate of 85%. This is decreased greatly in non-Hodgkin’s lymphoma where the survival rate is only 66% [97]. Overall, there has been progress in the treatment of lymphoma cancer subtypes, but improvements and advancements are still required.

There are a several treatment options that are commonly practiced. These include chemotherapy, biological therapy, targeted therapy, radiation therapy, stem cell transplant, and Chimeric antigen receptor (CAR) T-cell treatment. For starters, chemotherapy involves the use of drugs that quickly kill dividing cancer cells. The administration is often given orally through pill or tablet form, or through a catheter directly into the bloodstream. The side effects of chemotherapy depend on the dosage and specific drug that is taken. Some of the common side effects include hair loss, nausea, vomiting, loss of appetite, fatigue, increased bruising or bleeding, and a heightened chance of infection due to the loss of white blood cells [99]. Furthermore, biological therapy involves any treatment that utilizes living organisms, or elements from living organisms to treat cancer. This treatment works to strengthen the immune system in its recognition of irregular cells to then destroy them (ex. Vaccines). The side effects of biological therapies are found to be less severe than chemotherapy with some being rash, swelling, headaches, muscle aches, fevers, or fatigue [99]. Another common treatment option for chemotherapy is targeted therapy. This involves the use of drugs that disrupt a particular function of the cancerous cell instead of exhibiting general cytotoxic effects, which allows for less damage to normal than that found in other treatments such as chemotherapy. The side effects of targeted therapies can include swelling, bloating, diarrhea, vomiting and sudden weight gain [99]. Additionally, radiation therapy is a treatment option sometimes administered. It involves using radiation energy to target cancer cells. Radiation therapy is often used in the treatment of leukemia that has spread to the brain or spleen. The side effects depend on the location of the body that is exposed. Some possible side effects include nausea, vomiting and diarrhea [99]. These are seen in addition to the apparent damage of the skin where it will become red, dry and tender. In stem cell transplant, high doses of chemotherapy and/or radiation are used to terminate leukemia cells in addition to normal bone marrow. Afterwards, the transplanted stem cells are supplied. They travel to the bone marrow and start to make new blood cells. Despite the success of stem cell transplant, it comes with its own limitations. The transplantation requires patients to remain in the hospital for several weeks following the procedure. In this time or even up to years afterward, there is a possible risk in seeing an incidence of graft-versus-host disease (GVHD). In GVHD, the donor’s white blood cells react against the patient’s normal tissue. The effects range from mild to severe often involving the liver, skin, or digestive tract. Finally, chimeric antigen receptor (CAR) T-cell treatment is a newly formed option that takes a patient’s normal lymphocytes and alters them to attack leukemia cells and reinsert them into the patient’s blood. This treatment has only been used in individuals with B-cell lymphomas that perform stubbornly to other treatments. There are many side effects when it comes to this method. Some serious symptoms include fast heart rate, low blood pressure, nerve damage, and lowered immune function. Overall, these current treatment methods have shown some success in the management of leukemia and lymphoma, but with harmful side effects [99].

### 4.2. Anticancer Effects of Natural Compounds In-Vitro and In-Vivo

Natural health products are materials that have been isolated from particular foods and plant sources. Many have already shown effects in leukemia. For example, feverfew is a common garden flowering garden plant that is used in medicine. Several years ago, scientists were able to extract the compound parthenolide from feverfew and modify it to have anti-cancer effects. In addition, the plant Tripterygium wilfordii Hook.f. (TWHF) contains an active ingredient called triptolide. Triptolide has been shown in animal models (in vivo) to be effective against cancer, arthritis and skin graft rejection [100]. Moreover, blister beetles are a soft bodied plant eating beetle type. When disturbed, adult blister beetles release a defensive yellow oil known as cantharidin. It has been found to inhibit several types of cancer cells, leading to interest in the possible artificial production of this substance. Finally, veratrum caifornicum (corn lily) is a poisonous plant common in forested area in the Northwest and Pacific regions of the United States. The poisonous aspect are steroidal alkaloids, most notably cyclopamine [101]. The presence of cyclopamine in the corn lily was first brought to light in 1950, and today has the interest of researchers for its anti-cancerous properties in many animals. Overall, there are many natural compounds found to show anticancer effects both in vitro and in vivo.

### 4.3. Bioactive Compounds

**Figure 4:**
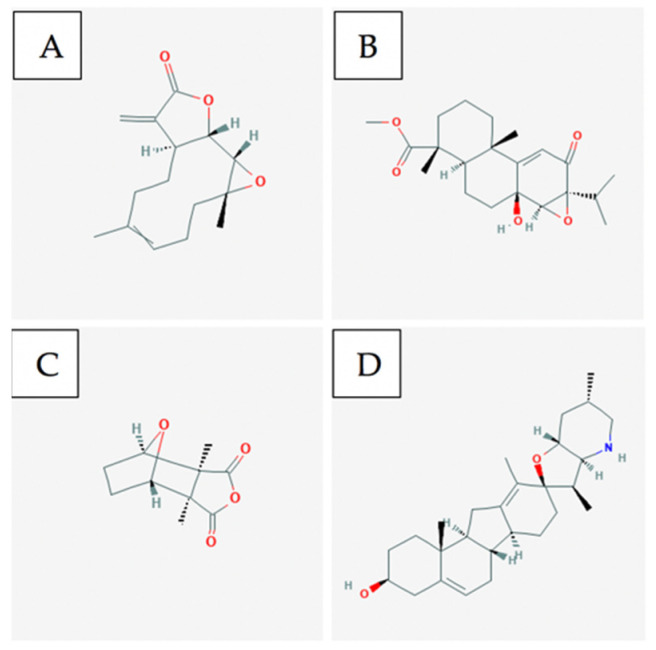
A few of the bioactive compounds with known anti-cancer activity towards leukemia and lymphoma: (**A**) Parthenolide; (**B**) Triptolide; (**C**) Cantharidin; (**D**) Cyclopamine [104,105,106,107].

Parthenolide (Figure 4A) is a sesquiterpene lactone that occurs naturally in the plant feverfew and has been shown to demonstrate anticancer properties. Parethenolide has been found to induce apoptosis in total as well as more of the phosphoglycoprotein marker CD34+ populations from human acute myeloid leukemia specimens [102]. Parthenolide was also shown to preferentially target AML (in vitro colony assay) in mice through inhibiting NF-kB, proapoptotic activation of tumour suppressor p53, and increased reactive oxygen species (ROS) production. It has also been reported that in MV4-11 leukemia cell lines, parethenolide displays inhibitory effects on DNMT1 activity linked to the alkylation of Cys1226 of DMT1, thus interfering with Sp1 which results in arrest at the G1 stage of the cell cycle [103].

Triptolide is a bioactive lipid and a traditionally used Chinese medicinal plant. Recent studies have shown that triptolide has a vast spectrum in anticancer activity toward various tumours resulting in the inhibition of tumour growth and inducing cell apoptosis. At lower doses, triptolide in combination with idarubicin is able to induce apoptosis in Leukemia stem cells in the KG1a cell line. Triptolide (Figure 4B) works through ROS regeneration and the downregulation of both the HIF1α and Nrf2 pathways [102].

Cantharidin (Figure 4C) is a natural toxin that is secreted by many species of blister beetles and is a promising compound for selectively targeting leukemia stem cells [108]. Cantharidin and its derivative Norcantharidin were found to inhibit hepatic leukemia factor, which is a gene overexpressed in leukemia stem cells. They are also able to specifically target acute myeloid leukemia by regulating the expression in pathways: SLUG, NFIL3, and c-myc, which prompt p53 and mitochondrial-caspase cascade to induce apoptosis [102].

Cyclopamine (Figure 4D) is a naturally occurring steroidal alkaloid that is isolated from the plant Veratrum californicum. In acute myeloid leukemia, cylopamine works to neutralize Hh ligands which results in Hh inhibition thus inducing apoptosis in CD34+ cell lines [109].

### 4.4. Single vs. Multiple Compounds and Collective Activities Targeting Multiple Pathways

Polyphenolic compounds commonly found in fruits and vegetables were evaluated for apoptotic effect in a hematological cell line. In one study, two polyphenolic compounds were evaluated for their anticancer effect. Ellagic acid (known to induce cell cycle arrest and apoptosis) and quercetin (known to inhibit cell cycle kinetics, proliferation, and induce apoptosis) were evaluated in combination for their anticancer effect on the MOLT-4 human leukemia cell line. The two compounds were found to interact synergistically, exhibiting an effect greater than the sum of their individual effects. Both compounds acted in synergy to induce apoptosis, decrease cell viability and mitochondrial activity, and activate caspase 3 [110]. A related study looked at the effects of a combination treatment of ellagic acid, quercetin, and resveratrol on the MOLT-4 human leukemia cell line. The results showed that all three compounds had a synergistic effect in the induction of apoptosis, and decrease in cell number and viability [111]. All of these compounds are found in muscadine grapes (Vitis rotundifolia), along with many other compounds that have various antioxidant and anticancer properties. Therefore, multiple compounds may work together to display synergistic activity to induce multiple anticancer effects compared with singular compounds.

## 5. Natural Health Products and Natural Compounds Used in the Treatment of Colorectal Cancer

### 5.1. Prevalence Statistics, Prognosis, and Downsides of Conventional Treatments

Colorectal cancer (CRC) is a heterogeneous disease that combines colon cancer and rectal cancer. CRC accounts for the fourth highest cancer mortality rate, corresponding to 608,000 deaths worldwide and approximately 1,234,000 new cases each year [112]. It is the second most commonly diagnosed cancer in females and the third in males [16]. Great advancements have been made in cancer research in terms of diagnosis. If CRC is diagnosed early, surgery has been a successful treatment with a high survival rate. However, once CRC advances to later stages, patients have fewer options left for treatment. The most popularly used conventional therapeutics for advanced-stage CRC include FOLFOX and 5-fluorouracil (5-FU). However, these drugs cause serious side-effects as a result of their non-selective targeting, which affects healthy cells as well [113]. Over many centuries, natural health products (NHPs) have shown to display anti-cancer activity by targeting multiple pathways in cancerous cells to selectively induce programmed cell death. Various NHPs contain polychemical mixtures that display potent anticancer activity and play a crucial role in the discovery and development of medication for the treatment of human diseases. For instance, dandelion root extract has shown anti-cancer properties by selectively triggering apoptotic pathways both in-vitro and in-vivo with CRC models [114]. Lemongrass extract and long pepper extract have also shown great potential as anticancer agents in CRC by inducing apoptosis in time- and dose-dependent manners without harming healthy cells [115,116,117]. The antitumour activity of rosemary extract has also been investigated, and results show that it strongly inhibited proliferation, migration, and colony formation of colorectal cancer cells [118]. Moreover, the relationship between curcumin and CRC has been investigated. In vitro studies performed on human CRC cell lines show that curcumin inhibited cellular growth and stimulated apoptosis, while in vivo studies displayed anti-carcinogenetic properties in inflammation-related and genetic CRC [119,120]. The scientific validation of these NHPs will be required to determine their efficacy, safety, and mechanisms of action. Furthermore, combined treatment options of two or more NHPs taken as supplements with chemotherapy should be investigated as multiple NHPs could signal more pathways activated in the body for cancer treatment.

Depending on the cancer stage and the degree of complication, contemporary treatment methods for CRC primarily rely on success of chemotherapy either alone or in a combination treatment with surgical resection or radiation therapy [121]. If detected early, the fatality risk associated with CRC can be minimized by surgical resection. When CRC develops into a malignant tumour and spreads throughout the body, it becomes a metastatic cancer known as an adenocarcinoma. Nearly half of the patients diagnosed with CRC progress to later stages of the disease, and advanced-stage CRC has limited treatment options [122]. The most common chemotherapeutics used for treatment of aggressive colon cancers include FOLFOX and Taxol. FOLFOX is a combination of folinic acid (leucovorin), 5-fluorouracil, and oxaliplatin. Although chemotherapeutic drugs are initially effective as they attack rapidly dividing cells, their non-selectivity towards both cancerous and normal, non-cancerous cells leads to serious side-effects in patients undergoing treatment [113,123]. These side-effects can include gastrointestinal issues and neurotoxicity [124].

### 5.2. Anticancer Effects of Natural Compounds In-Vitro and In-Vivo

NHPs have played a profound role in the discovery and development of cancer medication for centuries. Over 75% of the currently available chemotherapeutic drugs have been derived from natural sources, including plants, microbes, and marine sources [125]. Plant products especially contain many bioactive substances that have shown protective effects against several cancers, including colon cancer. However, these products receive little attention in biomedical research due to their lack of scientific credibility and experimental validation.

Long pepper, from the Piperaceae family, has been shown to selectively target a wide spectrum of cancer cells [116,117,126,127]. There are several compounds present in long pepper, including piperines and piperlongumines, that have demonstrated potent anticancer activity and exhibited roles in metabolic activation of carcinogens and mitochondrial energy production. A study specifically investigated the bioactive compound piperlongumine (PPLGM) found in long pepper and discovered that it inhibits the growth of colon cancer cells and selectively induces cell apoptosis in a time- and dose-dependent manner [126]. Further research will be required to understand the molecular mechanisms involved in PPLGM-mediated apoptosis to develop combination treatment strategies to treat colon cancer.

In recent decades, experimental research has demonstrated the pharmacological potential of rosemary and some of its primary compounds, including diterpenes carnosic acid (CA) and carnosol (CAR).

Curcumin is a yellow bioactive pigment compound naturally obtained from the rhizome of the Curcuma longa (turmeric) plant. It has anti-inflammatory, antioxidant, chemotherapeutic, anti-metastatic, and anti-angiogenic properties [128]. Due to curcumin’s preferential distribution in the colonic mucosa compared with other tissues, many initial clinical studies focus on identifying whether it may play a role in CRC models [129]. In-vitro studies performed on different human colon cancer cell lines show that curcumin significantly inhibited cell proliferation in a time- and dose-dependent manner through the induction of reactive oxygen species (ROS) generation and downregulation of E2F4 and related genes [122,130]. Mosieniak et al. also demonstrated that curcumin inhibited cell growth of colon cancer cells by interacting with multiple molecular targets, resulting in the modulation of several distinct signaling pathways [119]. In addition, in-vivo findings indicate that curcumin inhibits angiogenesis, which may indicate, in part, the antitumour activity of curcumin [131].

Additionally, an interesting finding has been reported recently by De Leso et al. where it was observed that the blockers AqB011 and bacopaside II exhibit synergistic action, amplifying the inhibition of 2D cell migration in two colon cancer cell lines [132].

### 5.3. Bioactive Compounds

**Figure 5:**
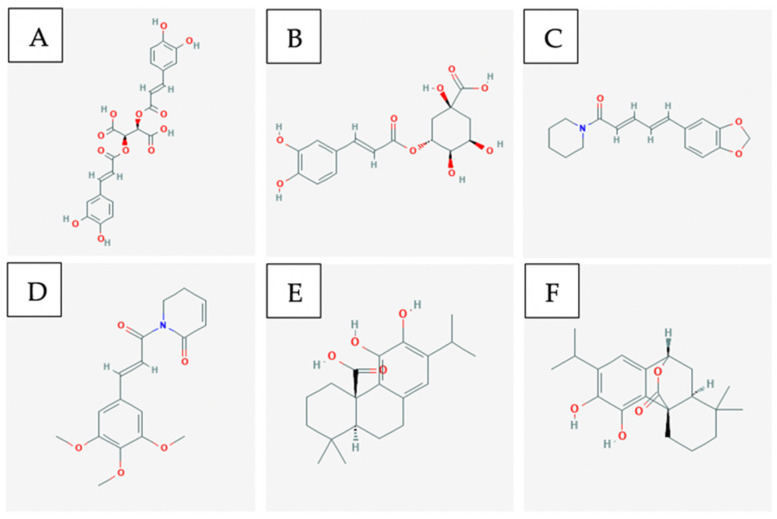
A few of the bioactive compounds with known anti-cancer activity towards colorectal cancer: (**A**) Chicoric acid; (**B**) Chlorogenic acid; (**C**) Piperine; (**D**) Piperlongumine; (**E**) Carnosic acid; (**F**) Carnosol [28,69,138,139,140,141].

Dandelion contains various phytochemicals including flavenoids, phenolic acids, alkaloids, and terpenes [129]. In recent years, researchers have become more interested in plant polyphenols due to their potent antioxidant properties and their influence in the prevention of various diseases, including cancer [133]. More than 30 phenolic compounds have been found and isolated in dandelion [134], with chicoric acid, chlorogenic acid (Figure 5A and B respectively), caffeic acid, and luteolin being the most abundant ones [135,136,137]. One study investigated compounds extracted from different parts of dandelion and found that dandelion leaf contains the highest antioxidant and 2,2-diphenyl-1-picrylhydrazyl (DPPH) scavenging activity; this is in agreement with its higher total phenolic content [135].

Lemongrass (LG) is known to have several biologically active compounds that have anti-mutagenic, anti-proliferative, and anti-parasitic properties [142,143]. A phytochemical composition analysis of LG determined that the constituents mostly belong to monoterpene, sesquiterpene, and phenolic acids [142].

Piper longum, which is one of the several species of long pepper, contains several classes of compounds including piperines, piperlongumine (PPLGM) (Figure 5C and D respectively), and dihydropiperlongumine [117]. Another study examined whether the c-Jun NH_2_-terminal (JNK) signaling pathway is involved in PPLGM-induced apoptosis in the human colon cancer cell line HCT116 [116]. JNKs are protein kinases that are believed to play an essential role in cancer cell death induced by redox chemotherapeutic agents [144,145]. Evidence from this study demonstrates that PPLGM-mediated apoptosis in the HCT116 cells is at least partially dependent on the activation of the JNK signal pathway, and PPLGM operated in a concentration- and time-dependent manner [116].

Rosemary is a bush of the Lamiaceae family that contains various phenolic compounds, with diterpenes carnosic acid (CA) and carnosol (CAR) (Figure 5E and F respectively) being the major ones. Carnosic acid poses antiproliferative activity in colon cancer cells [146], and carnosol demonstrates anti-inflammatory and anticancer activities on various cancer types, including colon cancer [147]. A study observed that CA both induces transcriptional activation of genes that encode detoxifying enzymes and alters the expression of genes traced with the transport and biosynthesis of terpenoids in the CRC line [148]. Functional analysis revealed that the activation of ROS metabolism and alteration of several genes involved in particular pathways may explain the transcriptional change induced by CA in HT-29 cells [148].

Curcumin is a hydrophobic polyphenol that is naturally obtained from the rhizome of the plant Curcuma longa, also known as turmeric [149]. A study found that curcumin induces the production of ROS and Ca^2+^, decreases the levels of mitochondria membrane potential, and induces caspase-3-activity [150]. Moreover, it was found to elevate the expression of Bax, cytochrome C, p53, and p21 [150]. One of the main mechanisms through which curcumin inhibits cell proliferation is the induction of apoptosis. A study revealed that curcumin blocked cell proliferation through the induction of ROS generation and downregulation of E2F4, cyclin A, p21, and p27 [122]. It was revealed that curcumin promoted apoptosis through the inhibition of NF-kB and by cell cycle arrest at the G2/M phase and partially the G1 phase [119,151]. Curcumin was also found to suppress growth and induce apoptosis in CRC via inhibition of hepatocyte growth factor receptor (c-MET), particularly the Sp transcription factor [152].

### 5.4. Single vs. Multiple Compounds and Collective Activities Targeting Multiple Pathways

Ovadje et al. conducted phytochemical analysis and bioassays of dandelion root that led to the identification of four pharmacologically active compounds: α-amyrin, β-amyrin, lupeol and taraxasterol [114]. They found that there were no synergistic interactions between these compounds, as anti-cancer activity was not displayed individually nor in combination treatments [114]. Another study examined the synergistic anti-inflammatory effects of luteolin and chicoric acid, two abundant constituents of dandelion [153]. Findings show that both compounds did not exhibit any synergistic activity, although both suppressed oxidative stress [153].

Lemongrass is composed of many compounds that interact in a complex manner. Philion et al. identified three unique compounds in the extract (elemicin, lonicerin, and methyl isoeugenol) and evaluated their anti-cancer activity, both individually and in combination [154]. The findings indicate that these compounds showed poor induction of apoptosis both alone and in combination, except with a combination of very high doses [154].

Sánchez et al. investigated the putative synergistic effects between the major compounds found in rosemary. The compounds bearing the highest antiproliferative activities, namely the diterpenes (CA and CAR) and the triterpenes betulinic acid (BA) and ursolic acid (UA) were examined in single treatments and in pairwise treatments [118]. Results indicate a dose-dependent antiproliferative effect of which the triterpenes BA and UA showed higher antiproliferative effect than the diterpenes CA and CAR [118]. Moreover, antagonism was observed for the BA-UA combination regardless of the treatment method used [118].

A study investigated the effects of a combination treatment of curcumin and 5-FU on the proliferation of an aggressive colon cancer cell line, HT-29 [155]. Their findings show synergistic inhibition of HT-29 growth, and this synergism was associated with a 6-fold decrease of COX-2 protein expression [155]. Another study investigated the effectiveness of the combination treatment of dasatinib (Src inhibitor) and curcumin in anti-proliferation of chemo-resistant colon cancer cells. Results demonstrate that the combinatorial therapy inhibited cellular growth, invasion, and colonosphere formation, suggesting that dasatinib and curcumin demonstrate a synergistic interaction and may present a therapeutic strategy [156]. Considering curcumin is poorly water soluble, extreme conditions of the intestinal tract can decrease its absorption. To overcome this drawback, Han et al. sought orally deliverable nanotherapeutics by combining curcumin and 7-ethyl-10-hydroxycamptothecin (SN38). This combination was found to exert synergistic beneficial effects on intestinal inflammation, and this could be attributed to the synergistic effect of SN38 and curcumin [157].

## 6. Natural Health Products, Natural Compounds, and Natural Extracts Used in the Treatment of Lung and Pancreatic Cancer

### 6.1. Prevalence Statistics, Prognosis, and Downsides of Conventional Treatments

In 2020, it is estimated that 6000 Canadians will be diagnosed with pancreatic cancer and 5300 will die as a result of the diagnosis. Out of the 6000, it is estimated that 3100 will be men and 2900 will be women [158]. In the United States, pancreatic cancer accounts for 3% of all cancers and 7% of all cancer deaths, with an average lifetime risk of 1 in 64. The 5-year relative survival rate, which measures the likelihood of survival relative to the population without the disease, is 37% for individuals with pancreatic cancer that has not metastasized outside the local area of the pancreas, drops to 3% for pancreatic cancer that has metastasized to distant organs such as the lungs, and overall, is 9% [159].

An estimated 29,800 Canadians will be diagnosed with lung cancer in 2020, representing 13% of all newly diagnosed cancer cases in 2020. In 2020, it is estimated that 21,200 Canadians will die from lung cancer, representing 25% of all cancer deaths. It is the most commonly diagnosed cancer (excluding non-melanoma skin cancer) and is the leading cause of death from cancer for both men and women in Canada [160]. Approximately 15,000 men and 14,800 women will be diagnosed with lung cancer in 2020, and 58 Canadians will die from lung cancer every day [160].

Pancreatic cancer is a grave disease with a high mortality rate as patients often remain asymptomatic until the disease progresses to more advanced stages [161]. Currently, surgical resection is the only curative form of treatment, and it is only viable for patients who have experienced no metastasis of the tumour [161]. After surgery is performed, the next standard of care is chemotherapy. Gemcitabine is a common chemotherapeutic and it is given in combination treatments with other drugs, such as erlotinib. However, clinical trials have shown that only patients who develop a rash after this treatment will experience significant survival benefits [161]. For patients in which the tumour has already metastasized, FOLFIRINOX (a multi-agent chemotherapeutic), is often used instead. However, it has side effects which include higher risks of neutropenia, neuropathy and gastrointestinal issues [161]. Gemcitabine along with albumin-bound paclitaxel is a more promising chemotherapeutic treatment for patients dealing with metastatic pancreatic cancer, but not all patients can tolerate it [161]. Chemoradiotherapy has not been shown to be significantly better than chemotherapy alone for patients with localized pancreatic cancer [161]. Immunotherapy has also been recently investigated as a potential treatment. So far, vaccines and immune checkpoint inhibitors developed against pancreatic cancer have not fared well, but the potential of adoptive T-cell transfer should be investigated further [162].

The treatment regimen administered for patients dealing with lung cancer are of particular importance due to its poor prognosis. There are two variations of this disease: the more common non-small cell lung cancer, and the less common small cell lung cancer, which is more aggressive. For stages I, II and III of non-small cell lung cancer, surgical procedures are used to remove the tumours [163]. For more advanced stages of this disease, a chemotherapeutic regimen of platinum agents such as cisplatin or carboplatin along with paclitaxel, gemcitabine, docetaxel, vinorelbine, irinotecan or pemetrexed is given. However, there is a risk of toxicity, especially for patients with low performance status [163]. Radiotherapy, including stereotactic body radiation therapy, can be effective, but only for patients who have localized lung cancer [163]. Immunotherapy has shown mixed success as tumour vaccine development has been ineffective, but antibody-directed therapies against inhibitory checkpoint molecules have been promising, especially for the advanced stages of lung cancer [164]. Small cell lung cancer tumours are more aggressive, as mentioned above, but are also more sensitive to both chemotherapies and radiotherapies. However, relapse is very common for patients [165].

### 6.2. Anticancer Effects of Natural Compounds In Vitro and In Vivo

Curcumin is a major component of the turmeric plant [166]. Curcumin affects pancreatic cancer cells by preventing the development and metastasis of pancreatic tumours [167]. Capsaicin is a compound obtained from chilli peppers and it has demonstrated anti-cancer activity in both in vitro and in vivo tests of xenografted mice when the human pancreatic cell line PANC-1 was used [168]. Several flavonoids, which are natural plant pigments, have also shown anti-cancer activity against pancreatic cancer cells including: quercetin which is found in fruits and vegetables, epigallocatechin-3-gallate which is found in in green tea, and genistein which is found in soy [167]. Ginsenosides are another example of anti-cancer natural compounds and are found within ginseng, a traditional Chinese medicine which induces apoptosis in pancreatic cancer cells [167].

Similarly, many natural compounds have been identified as anti-cancer agents for lung cancer. This includes Saikosaponin D, a major anti-cancer constituent of the Bupleurum plant [169]. This compound has been used in traditional Chinese medicine and has been shown to inhibit cell proliferation in the A459 lung cancer cell line [169]. Flavonoids again appear as anti-cancer natural compounds due to their strong antioxidant effects. Particularly, isoliquiritigenin has shown strong activity against the A549 lung cancer cell line [170].

### 6.3. Bioactive Compounds

**Figure 6:**
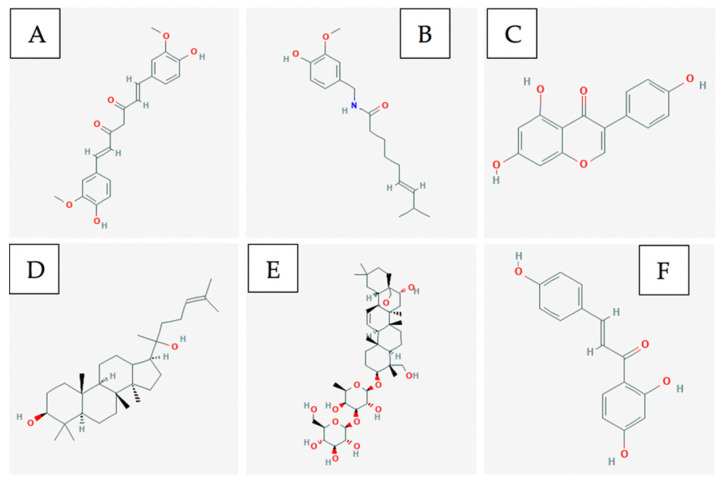
A few of the bioactive compounds with known anti-cancer activity towards lung and pancreatic cancer: (**A**) Curcumin; (**B**) Capsaicin; (**C**) Genistein; (**D**) Ginsenosides; (**E**) Saikosaponin D; (**F**) Isoliquiritigenin [31,171,172,173,174,175].

Curcumin (Figure 6A) is the major anti-cancer agent within the turmeric spice. It inhibits pancreatic cancer cell growth by slowing down the phosphorylation of two important signalling pathways for tumour development, STAT3 and AKT [167]. Another pathway it affects to prevent pancreatic cancer cell proliferation is the NF-kB pathway; this prevents downstream signals such as COX-2 from being activated [167]. Since it is not bioavailable in the body, fluorocurcumin analogs are often used instead [167].

Capsaicin (Figure 6B) is a compound found within red chilli peppers which causes apoptosis in pancreatic cancer cells by down-regulating Bcl-2 and survivin protein levels [167]. In addition, it leads to cytochrome c release from the mitochondria which activates other apoptotic factors [167].

Genistein (Figure 6C) is an isoflavone that can be obtained from soy-based products and it primarily functions as a tyrosine kinase inhibitor [167]. It has been shown to induce apoptosis in pancreatic cancer cells by regulating the activity of STAT3, AKT, NF-kB and down-regulating Notch-1 [167]. 

Ginsenosides (Figure 6D), also commonly referred to as triterpene glycosides, it is a major bio-active compound found within the ginseng plant. It induces apoptosis in pancreatic cancer cells by down-regulating Bcl-2 and survivin protein while up-regulating p53 and Bax protein [167].

Saikosaponin D (Figure 6E) is one of the common triterpene saponins found within plants of the Bupleurum genus and it displays anti-inflammatory, immunomodulatory and anti-viral effects [169]. The exact mechanism of this compound is unknown for pancreatic cancer cells, but it is p53- and caspase-dependent [169].

Isoliquiritigenin (Figure 6F) is an example of a flavonoid that can be found within licorice and shallots; it displays anti-inflammatory and antiplatelet aggregation properties [170]. It affects lung cancer cells by causing cell cycle arrest in the G1 phase through the upregulation of p53 and p21/WAF1 proteins [170]. It also induces apoptosis by up-regulating Fas and its two ligands: membrane-bound Fas ligand and soluble Fas ligand [170].

### 6.4. Single vs. Multiple Compounds and Collective Activities Targeting Multiple Pathways

The Curcuma longa plant is primarily composed of curcuminoids and essential oils [176]. The primary curcuminoid is curcumin which displays anti-cancer activities through the anti-oxidative and anti-inflammatory pathways mentioned above, but other curcuminoids such as demethoxycurcumin and bisdemethoxycurcumin exist within this plant as well [176,177]. The active ingredients in the essential oil include ar-turmerone, α-turmerone, and β-turmerone which also show anti-cancer effects through anti-inflammatory and anti-oxidative pathways [176].

The root of the Panax ginseng plant has two primary bio-active ingredients which includes ginsenoside and gitonin [178]. As mentioned above, ginsenosides can target pancreatic cancer cells by regulating the levels of several proteins involved in apoptotic pathways. However, evidence suggests that gitonin could also potentially have an anti-cancer effect as it has been shown to inhibit autotaxin activity which is a molecule required for metastasis to occur in many tumours [178]. Further studies must be done to assess the effect of gitonin on pancreatic cancer cells specifically.

The roots of the Bupleurum radix plant has two natural compounds which includes Saikosaponin-a (SSa) and Saikosaponin -d (SSd) [169]. SSa and SSd have both been shown to have an anti-cancer effect by inducing pathways that increase the amount of ROS within cancer cells; both have displayed caspase dependent activity [169].

## 7. Natural Health Products and Natural Compounds Used in the Treatment of Prostate Cancer

### 7.1. Prevalence Statistics, Prognosis, and Downsides of Conventional Treatments

In more developed countries, prostate cancer is the leading cause of death among males [179]. In 2019 alone, there were 174,650 estimated new prostate cancer cases in the United States, with 31,620 estimated deaths. Prostate, lung and colorectal cancer are the cause of 42% of male cancer diagnoses. Prostate cancer is responsible for about one in five new cases [180].

In order to manage this type of cancer, there are different treatment options available. It is possible that many patients will not benefit from any treatment and may just need active surveillance. This type of treatment is usually used on patients when the cancer is not likely to progress or has a low grade/stage level. Active surveillance can also reduce adverse side effects associated with certain treatments. There are 60–70% of males that show some areas of cancer in their prostate, with a lot of them only needing active surveillance. This sort of treatment is also used for males that have a shorter life expectancy. In the case of longer life expectancy and a higher grade of the cancer, treatments like surgery and radiation may then be needed [181].

It is possible to have the cancer in only a small portion of the prostate, which is usually very easy to treat and may not need treatment if there is no risk towards the patient. There have been improvements in technology for the treatment of this disease. There is now more advanced imaging, biopsy methodology, and understanding of the factors that are causing the tumours. Specifically, procedures such as interstitial prostate brachytherapy (IPB), external beam radiotherapy, radical prostatectomy (RP), cryotherapy, hormonal treatments, and chemotherapy have been used to treat varying stages of prostate cancer [181].

The several different treatment options available for prostate cancer come with risks and difficulties. On the surgical side, the use of RP on high-risk patients has been associated with complications such as lymph node metastasis, early loss of erection, and urinary incontinence. Difficulties with radiation therapies include painful urination, inflammation, damage to the rectum, and rectal bleeding. With regards to chemotherapy, they have not been found to be very effective towards prostate cancer. Certain chemotherapeutic drugs, such as mitoxantrone with prednisone, will improve quality of life and pain of the patient but are not very effective for survival [182]. Chemotherapy can also cause systemic toxicity, harming healthy cells in the body as well as the cancer cells [181].

### 7.2. Bioactive Compounds

**Figure 7:**
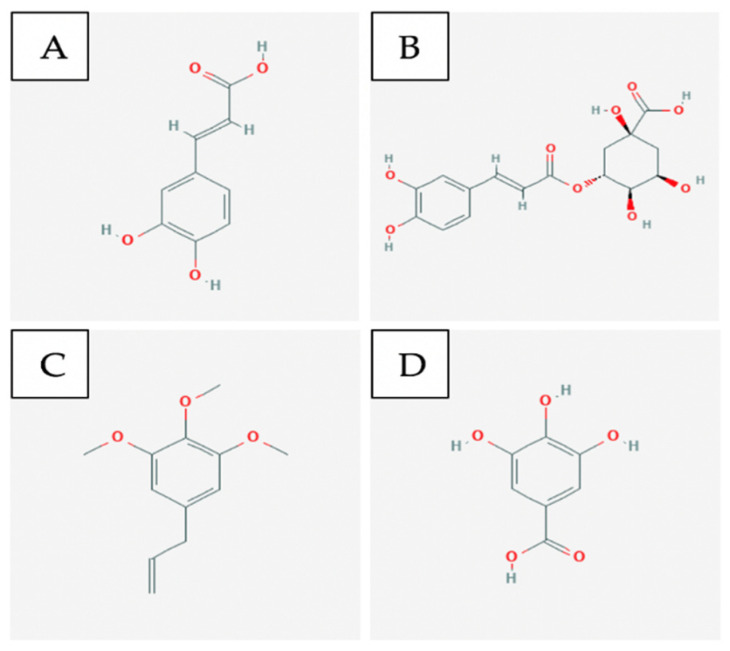
A few of the bioactive compounds with known anti-cancer activity towards prostate cancer: (**A**) Caffeic acid; (**B**) Chlorogenic acid; (**C**) Elemicin; (**D**) Gallic acid [139,185,186,187].

Major components of dandelion root include caffeic and chlorogenic acids (Figure 7A and B respectively) along with vitexin-2-rhamnoside (apigenin). These compounds have been shown to be strong antioxidants while having anti-inflammatory properties [183,184].

There have been three major components identified in lemongrass—elemicin (Figure 7C), lonicerin, and methyl isoeugenol. These compounds have shown poor anti-cancer efficacy when used individually [156].

Major components of Muscadine grapes are polyphenol constituents such as gallic acid (Figure 7D), ellagic acid glucosides, catechin, quercetin, kaempferol, and several anthocyanins [188]. Plant polyphenols have been shown to have antiproliferative and anti-apoptotic properties [189].

### 7.3. Single vs. Multiple Compounds and Collective Activities Targeting Multiple Pathways

Dandelion Root can be resolved into many compounds, such as caffeic and chlorogenic acids. Caffeic and chlorogenic acids have been shown to hamper the development of metastatic cancer [190]. Apigenin is another compound found in this extract that has been shown to reduce prostate cancer stem cell survival and migration through downregulation of PI3K/Akt/NF-κB signaling [191].

Elemicin, lonicerin, and methyl isoeugenol are the major compounds found in lemongrass. The induction of glutathione S-transferase is believed to be the major mechanism for chemical carcinogen detoxification by these compounds [192]. Flavonoids, including gallic acid, isoquercetin, quercetin, rutin, catechin and tannic acid, are also found in lemongrass. Quercetin has been shown to act as a chemopreventive agent in vivo studies by increasing antioxidant enzymes and apoptotic proteins that were significantly decreased in cancer-induced animal models [193].

The polyphenol components in Muscadine grapes were shown to decrease the expression of AKT, which is a client protein heat shock protein (Hsp) involved in the signal transduction of cancerous prostate cell growth. Hsp40 was targeted by these compounds leading to a significant reduction in growth and migration of prostate cancer cells [194].

## 8. Conclusions and Future Prospects

Despite the significant progress in validating NHPs that have been made in recent years, several knowledge gaps remain that hinder their consideration as breast-cancer treatments in the scientific community. Notably, herb–drug interactions are an important field of study that has received little attention. Although several of the individual compounds have had their mechanisms clearly defined, entire extracts have seldom had them studied. Since little is known about an extract’s mechanism, it is difficult to speak about the possible synergistic effect that the compounds may display when administered together.

In reference to the several clinical trials that have been conducted, only a select few were done using the golden standard for establishing scientific evidence: a double-blind randomized clinical trial. Several of the clinical trials based their results on subjective data such as self-reported questionnaires. We suggest that future projects focus on extract characterization, the elucidation of their mechanisms, and on the herb–drug interactions in order to further validate NHPs as candidates for clinical trials against breast cancer.

As a result of this review of the literature, it is evident that there are some promising NHPs being researched for use in melanoma. For example, curcumin and its analogs have shown potential in preclinical models over the past decade [39,53,93] and it may be possible that it follows a similar trajectory as vincristine or vinblastine as a natural product. In addition, compounds found within traditionally used extracts such as berberine have shown validated anti-cancer activity against this particularly aggressive skin cancer. Although it is clear that there is considerable scientific validation towards NHPs to be used in melanoma, future preclinical research should be conducted on screening effective compounds for anti-cancer activity, particularly those with the potential to inhibit NF-κB as it has shown to be a protein involved in the ability for melanoma tumours to proliferate and avoid apoptosis [195]. Furthermore, there is limited information available towards the combinatorial use of NHPs alongside standard melanoma treatments. In particular, there should be a focus towards dacarbazine, the standard single-agent chemotherapy for melanoma, immunotherapies, and targeted therapies for BRAF/MEK mutations, as the latter two have emerged within the past decade as promising treatments. With the substantial preclinical data available for the combination of NHPs with clinically relevant therapies, clinicians and researchers may be more motivated to begin the study of evidence-based complementary treatments in clinical trials. Thus, we believe that further scientific research into validating new and current NHPs can assist in closing the clear gap that is seen with the current state of limited clinical data.

Further research is required to elucidate the nature of interactions between the phytochemical constituents present in NHP plant-based extracts. There have been promising studies showing synergistic biochemical interactions between compounds that make up extracts [110,111]. Since natural extracts are often complex in their phytochemical make-up, the primary anticancer compounds within the extract need to be characterized, isolated, and studied for further elucidation into their mechanism of action [196].

Cellular, animal, and clinical studies done so far indicate that various natural extracts and their constituent phytochemicals can serve as supplemental therapy to aid in the efficacy of treatment of individuals with colorectal cancer. However, there still needs to be further research done to elucidate drug–drug interactions between natural extracts/NHP and chemotherapy drugs. Furthermore, more phase I and II clinical trials are required to establish the toxicity, confirm efficacy, and evaluate the best guidelines for the development of various NHP’s as complementary or supplemental therapy [197].

More phase I and II clinical trials are required for NHP’s that have shown an anticancer effect in cellular and animal models. Furthermore, progression to later stages of clinical testing is required in the few NHP therapeutics that have shown promising results in phase I and II trails, such as the PHY906 botanical extract evaluated at yale cancer center in patients with advanced pancreatic cancer [198,199]. Those clinical trials also brought attention to natural extracts and preparations with multiple herbs. These extractions have the ability to induce synergistic effects in combination with conventional treatments, such as chemotherapeutics, while reducing the toxicity of the treatment regimen [199]. This ability of natural extracts to act via multiple pathways to induce various anticancer effects explains why they are able to increase efficacy and bioavailability in combination with conventional therapy, while simultaneously decreasing toxicity. This is especially important in cancer subtypes such as pancreatic cancer which is hard to treat and remains the most lethal form of cancer [200]. Clinical trials must also evaluate the levels of biomarkers of cancerous disease progression such as NF-kB transcription levels and cytokine levels to provide elucidation and validity to clinical results. This can also lead to identification of therapeutic targets to deal with symptoms and side-effects experienced by patients [199].

## Abbreviations

NHP	Natural Health Products
CT	Chemotherapy
RT	Radiation therapy
CAM	Complementary and alternative medicine
CRC	Colorectal cancer
DRE	Dandelion root extract
SERM	Selective estrogen receptor modulator
ER+	Estrogen-receptor positive cell
TNBC	Triple negative breast cancer cell
ATP	Adenosine triphosphate
CSC	Cancer stem cell
AMPK	AMP-activated protein kinase
ERK	Extracellular signal-related kinase
ERV-EA	Epstein-Barr virus early antigen
EMT	Epithelial-mesenchymal transitions
DNMT1	DNA Methyltransferase 1
FAK	Focal adhesion kinase
VEGF	Vascular endothelial growth factor
PST	Pancratistatin
TAM	Tamoxifen
ROS	Reactive oxygen species
NHL	Non-Hodgkin’s lymphoma
CAR	Chimeric antigen receptor
AML	Acute myeloid leukemia
FOLFOX	Combination Chemotherapy drug: Consists of folinic acid, 5-FU, and oxaliplatin
5-FU	5-fluorouracil
PPLGM	Piperlongumine
CA	Carnosic acid
CAR	Carnosol
JNK	c-Jun N-terminal kinase
BA	Betulinic acid
UA	Ursolic acid
SSa	Saikosaponin-a
SSd	Saikosaponin-d
Hsp	Heat shock protein

## References

[1] GBD Results Tool|GHDx. http://ghdx.healthdata.org/gbd-results-tool.

[2] Adjuvant Therapy: Treatment to Keep Cancer from Returning-Mayo Clinic. https://www.mayoclinic.org/diseases-conditions/cancer/in-depth/adjuvant-therapy/art-20046687.

[3] Mayer E.L. (2013). Early and Late Long-Term Effects of Adjuvant Chemotherapy. Am. Soc. Clin. Oncol. Educ. Book.

[4] Partridge A.H., Burstein H.J., Winer E.P. (2001). Side Effects of Chemotherapy and Combined Chemohormonal Therapy in Women with Early-Stage Breast Cancer. JNCI Monogr..

[5] Stone J.B., DeAngelis L.M. (2016). Cancer-treatment-induced neurotoxicity-focus on newer treatments. Nat. Rev. Clin. Oncol..

[6] Complementary, Alternative, or Integrative Health: What’s in a Name?|NCCIH. https://www.nccih.nih.gov/health/complementary-alternative-or-integrative-health-whats-in-a-name.

[7] Garland S.N., Valentine D., Desai K., Li S., Langer C., Evans T., Mao J.J. (2013). Complementary and alternative medicine use and benefit finding among cancer patients. J. Altern. Complement. Med..

[8] Nutritional Assessment and Use of Complementary and Alternative Medicine in...: EBSCOhost. http://web.b.ebscohost.com.ledproxy2.uwindsor.ca/ehost/pdfviewer/pdfviewer?vid=1&sid=f8bac6c0-449c-4aec-800d-83db0c25dd19%40pdc-v-sessmgr03.

[9] Paul M., Davey B., Senf B., Stoll C., Münstedt K., Mücke R., Micke O., Prott F.J., Buentzel J., Huebner J. (2013). Patients with advanced cancer and their usage of complementary and alternative medicine. J. Cancer Res. Clin. Oncol..

[10] Complementary and Alternative Medicine (CAM)-National Cancer Institute. https://www.cancer.gov/about-cancer/treatment/cam.

[11] Dasgupta A., Bernard D.W. (2006). Herbal Remedies Effects on Clinical Laboratory Tests.

[12] Comelli M.C., Mengs U., Schneider C., Prosdocimi M. (2007). Toward the definition of the mechanism of action of silymarin: Activities related to cellular protection from toxic damage induced by chemotherapy. Integr. Cancer Ther..

[13] Luo Q., Asher G.N. (2017). Complementary and Alternative Medicine Use at a Comprehensive Cancer Center. Integr. Cancer Ther..

[14] Golden E.B., Lam P.Y., Kardosh A., Gaffney K.J., Cadenas E., Louie S.G., Petasis N.A., Chen T.C., Schönthal A.H. (2009). Green tea polyphenols block the anticancer effects of bortezomib and other boronic acid-based proteasome inhibitors. Blood.

[15] Sparreboom A., Cox M.C., Acharya M.R., Figg W.D. (2004). Herbal remedies in the United States: Potential adverse interactions with anticancer agents. J. Clin. Oncol..

[16] Bray F., Ferlay J., Soerjomataram I., Siegel R.L., Torre L.A., Jemal A. (2018). Global cancer statistics 2018: GLOBOCAN estimates of incidence and mortality worldwide for 36 cancers in 185 countries. CA Cancer J. Clin..

[17] Kolak A., Kamińska M., Sygit K., Budny A., Surdyka D., Kukiełka-Budny B., Burdan F. (2017). Primary and secondary prevention of breast cancer. Ann. Agric. Environ. Med..

[18] Reinert T., de Paula B., Shafaee M.N., Souza P.H., Ellis M.J., Bines J. (2018). Endocrine therapy for ER-positive/HER2-negative metastatic breast cancer. Chin. Clin. Oncol..

[19] Lumachi F., Brunello A., Maruzzo M., Basso U., Basso S.M.M. (2013). Treatment of estrogen receptor-positive breast cancer. Curr. Med. Chem..

[20] Liedtke C., Mazouni C., Hess K.R., André F., Tordai A., Mejia J.A., Symmans W.F., Gonzalez-Angulo A.M., Hennessy B., Green M. (2008). Response to Neoadjuvant Therapy and Long-Term Survival in Patients with Triple-Negative Breast Cancer. J. Clin. Oncol..

[21] Maughan K.L., Lutterbie M.A., Ham P. (2010). Treatment of Breast Cancer. Am. Fam. Physician.

[22] Padma V.V. (2015). An overview of targeted cancer therapy. BioMedicine.

[23] Nguyen C., Baskaran K., Pupulin A., Ruvinov I., Zaitoon O., Grewal S., Scaria B., Mehaidli A., Vegh C., Pandey S. (2019). Hibiscus flower extract selectively induces apoptosis in breast cancer cells and positively interacts with common chemotherapeutics. BMC Complement. Altern. Med..

[24] Wong R.S. (2011). Apoptosis in cancer: From pathogenesis to treatment. J. Exp. Clin. Cancer Res..

[25] De Souza Grinevicius V.M.A., Kviecinski M.R., Santos Mota N.S.R., Ourique F., Porfirio Will Castro L.S.E., Andreguetti R.R., Gomes Correia J.F., Filho D.W., Pich C.T., Pedrosa R.C. (2016). Piper nigrum ethanolic extract rich in piperamides causes ROS overproduction, oxidative damage in DNA leading to cell cycle arrest and apoptosis in cancer cells. J. Ethnopharmacol..

[26] Greenshields A.L., Doucette C.D., Sutton K.M., Madera L., Annan H., Yaffe P.B., Knickle A.F., Dong Z., Hoskin D.W. (2015). Piperine inhibits the growth and motility of triple-negative breast cancer cells. Cancer Lett..

[27] Burande A.S., Viswanadh M.K., Jha A., Mehata A.K., Shaik A., Agrawal N., Poddar S., Mahto S.K., Muthu M.S. (2020). EGFR Targeted Paclitaxel and Piperine Co-loaded Liposomes for the Treatment of Triple Negative Breast Cancer. AAPS PharmSciTech.

[28] Piperine|C17H19NO3-PubChem. https://pubchem.ncbi.nlm.nih.gov/compound/Piperine.

[29] 1,3-Pentadiene|C5H8-PubChem. https://pubchem.ncbi.nlm.nih.gov/compound/1_3-Pentadiene#section=2D-Structure.

[30] Betulin|C30H50O2-PubChem. https://pubchem.ncbi.nlm.nih.gov/compound/Betulin.

[31] Curcumin|IC21H20O6-PubChem. https://pubchem.ncbi.nlm.nih.gov/compound/Curcumin#section=2D-Structure.

[32] Lin H.-H., Chen J.-H., Kuo W.-H., Wang C.-J. (2007). Chemopreventive properties of Hibiscus sabdariffa L. on human gastric carcinoma cells through apoptosis induction and JNK/p38 MAPK signaling activation. Chem. Biol. Interact..

[33] Amran N., Rani A.A., Mahmud R., Yin K. (2016). Antioxidant and cytotoxic effect of Barringtonia racemosa and Hibiscus sabdariffa fruit extracts in MCF-7 human breast cancer cell line. Pharmacogn. Res..

[34] Afiune L.A.F., Leal-Silva T., Sinzato Y.K., Moraes-Souza R.Q., Soares T.S., Campos K.E., Fujiwara R.T., Herrera E., Damasceno D.C., Volpato G.T. (2017). Beneficial effects of Hibiscus rosa-sinensis L. flower aqueous extract in pregnant rats with diabetes. PLoS ONE.

[35] Bhaskar A., Nithya V. (2011). Phytochemical Screening and In Vitro Antioxidant Activities of the Ethanolic Extract of Hibiscus Rosa Sinensis L.. Sch. Res. Libr. Ann. Biol. Res..

[36] Hsu R.-J., Hsu Y.-C., Chen S.-P., Fu C.-L., Yu J.-C., Chang F.-W., Chen Y.-H., Liu J.-M., Ho J.-Y., Yu C.-P. (2015). The triterpenoids of Hibiscus syriacus induce apoptosis and inhibit cell migration in breast cancer cells. BMC Complement. Altern. Med..

[37] Syed Najmuddin S.U.F., Romli M.F., Hamid M., Alitheen N.B., Nik Abd Rahman N.M.A. (2016). Anti-cancer effect of Annona Muricata Linn Leaves Crude Extract (AMCE) on breast cancer cell line. BMC Complement. Altern. Med..

[38] Zafra-Polo M.C., González M.C., Estornell E., Sahpaz S., Cortes D. (1996). Acetogenins from annonaceae, inhibitors of mitochondrial complex I. Phytochemistry.

[39] Parashar K., Sood S., Mehaidli A., Curran C., Vegh C., Nguyen C., Pignanelli C., Wu J., Liang G., Wang Y. (2019). Evaluating the Anti-cancer Efficacy of a Synthetic Curcumin Analog on Human Melanoma Cells and Its Interaction with Standard Chemotherapeutics. Molecules.

[40] Pignanelli C., Ma D., Noel M., Ropat J., Mansour F., Curran C., Pupulin S., Larocque K., Wu J., Liang G. (2017). Selective Targeting of Cancer Cells by Oxidative Vulnerabilities with Novel Curcumin Analogs. Sci. Rep..

[41] Nakhjavani M., Palethorpe H.M., Tomita Y., Smith E., Price T.J., Yool A.J., Pei J.V., Townsend A.R., Hardingham J.E. (2019). Stereoselective anti-cancer activities of ginsenoside rg3 on triple negative breast cancer cell models. Pharmaceuticals.

[42] Nakhjavani M., Hardingham J.E., Palethorpe H.M., Tomita Y., Smith E., Price T.J., Townsend A.R. (2019). Ginsenoside Rg3: Potential Molecular Targets and Therapeutic Indication in Metastatic Breast Cancer. Medicines.

[43] Mai T.T., Moon J.Y., Song Y.W., Viet P.Q., Van Phuc P., Lee J.M., Yi T.H., Cho M., Cho S.K. (2012). Ginsenoside F2 induces apoptosis accompanied by protective autophagy in breast cancer stem cells. Cancer Lett..

[44] Kang J.H., Song K.H., Woo J.K., Park M.H., Rhee M.H., Choi C., Oh S.H. (2011). Ginsenoside Rp1 from Panax ginseng Exhibits Anti-cancer Activity by Down-regulation of the IGF-1R/Akt Pathway in Breast Cancer Cells. Plant Foods Hum. Nutr..

[45] Palethorpe H.M., Smith E., Tomita Y., Nakhjavani M., Yool A.J., Price T.J., Young J.P., Townsend A.R., Hardingham J.E. (2019). Bacopasides I and II act in synergy to inhibit the growth, migration and invasion of breast cancer cell lines. Molecules.

[46] Brenner D.R., Weir H.K., Demers A.A., Ellison L.F., Louzado C., Shaw A., Turner D., Woods R.R., Smith L.M. (2020). Projected estimates of cancer in Canada in 2020. CMAJ.

[47] Sandru A., Voinea S., Panaitescu E., Blidaru A. (2014). Survival rates of patients with metastatic malignant melanoma. J. Med. Life.

[48] Rosenberg S.A. (1976). Cancer Treatment Reports-Google Books.

[49] Jiang G., Li R.H., Sun C., Liu Y.Q., Zheng J.N. (2014). Dacarbazine combined targeted therapy versus dacarbazine alone in patients with malignant melanoma: A meta-analysis. PLoS ONE.

[50] Selby M., Engelhardt J., Lu L.-S., Quigley M., Wang C., Chen B., Korman A.J. (2013). Antitumor activity of concurrent blockade of immune checkpoint molecules CTLA-4 and PD-1 in preclinical models. J. Clin. Oncol..

[51] Chinembiri T.N., Du Plessis L.H., Gerber M., Hamman J.H., Du Plessis J. (2014). Review of natural compounds for potential skin cancer treatment. Molecules.

[52] Chatterjee S.J., McNulty J., Pandey S. (2011). Sensitization of human melanoma cells by tamoxifen to apoptosis induction by pancratistatin, a nongenotoxic natural compound. Melanoma Res..

[53] Bush J.A., Cheung K.J.J., Li G. (2001). Curcumin induces apoptosis in human melanoma cells through a Fas receptor/caspase-8 pathway independent of p53. Exp. Cell Res..

[54] Jordan M.A., Himes R.H., Wilson L. (1985). Comparison of the Effects of Vinblastine, Vincristine, Vindesine, and Vinepidine on Microtubule Dynamics and Cell Proliferation in Vitro. Cancer Res..

[55] Leung G.P., Feng T., Sigoillot F.D., Geyer F.C., Shirley M.D., Ruddy D.A., Rakiec D.P., Freeman A.K., Engelman J.A., Jaskelioff M. (2019). Hyperactivation of MAPK Signaling Is Deleterious to RAS/RAF-mutant Melanoma. Mol. Cancer Res..

[56] Soengas M.S., Lowe S.W. (2003). Apoptosis and melanoma chemoresistance. Oncogene.

[57] Zigler M., Villares G.J., Lev D.C., Melnikova V.O., Bar-Eli M. (2008). Tumor Immunotherapy in Melanoma. Am. J. Clin. Dermatol..

[58] Kalal B.S., Upadhya D., Pai V.R. (2017). Chemotherapy resistance mechanisms in advanced skin cancer. Oncol. Rev..

[59] Lesueur P., Lequesne J., Barraux V., Kao W., Geffrelot J., Grellard J.M., Habrand J.L., Emery E., Marie B., Thariat J. (2018). Radiosurgery or hypofractionated stereotactic radiotherapy for brain metastases from radioresistant primaries (melanoma and renal cancer). Radiat. Oncol..

[60] Marconcini R., Spagnolo F., Stucci L.S., Ribero S., Marra E., De Rosa F., Picasso V., Di Guardo L., Cimminiello C., Cavalieri S. (2017). Current status and perspectives in immunotherapy for metastatic melanoma. Oncotarget.

[61] Andrews S., Holden R. (2012). Characteristics and management of immune-related adverse effects associated with ipilimumab, a new immunotherapy for metastatic melanoma. Cancer Manag. Res..

[62] Nguyen C., Mehaidli A., Baskaran K., Grewal S., Pupulin A., Ruvinov I., Scaria B., Parashar K., Vegh C., Pandey S. Dandelion Root and Lemongrass Extracts Induce Apoptosis, Enhance Chemotherapeutic Efficacy, and Reduce Tumour Xenograft Growth In Vivo in Prostate Cancer. https://www.hindawi.com/journals/ecam/2019/2951428/?fbclid=IwAR0EXxzbbdw5M2y_GjMAHxwo9X0MyVBItBRDpO04_0uTLebIbonAfk60w6M.

[63] Alqathama A., Prieto J.M. (2015). Natural products with therapeutic potential in melanoma metastasis. Nat. Prod. Rep..

[64] Mittal A., Tabasum S., Singh R.P. (2014). Berberine in combination with doxorubicin suppresses growth of murine melanoma B16F10 cells in culture and xenograft. Phytomedicine.

[65] Noble R.L. (1990). The discovery of the vinca alkaloids-Chemotherapeutic agents against cancer. Biochem. Cell Biol..

[66] Legha S.S., Ring S., Papadopoulos N., Plager C., Chawla S., Benjamin R. (1989). A Prospective Evaluation of a Triple-Drug Regimen Containing Cisplatin, Vinblastine, and Dacarbazine (CVD) for Metastatic Melanoma. Cancer.

[67] Sawada N., Kataoka K., Kondo K., Arimochi H., Fujino H., Takahashi Y., Miyoshi T., Kuwahara T., Monden Y., Ohnishi Y. (2004). Betulinic acid augments the inhibitory effects of vincristine on growth and lung metastasis of B16F10 melanoma cells in mice. Br. J. Cancer.

[68] Aggarwal B.B., Yuan W., Li S., Gupta S.C. (2013). Curcumin-free turmeric exhibits anti-inflammatory and anticancer activities: Identification of novel components of turmeric. Mol. Nutr. Food Res..

[69] Piperlongumine|C17H19NO5-PubChem. https://pubchem.ncbi.nlm.nih.gov/compound/Piperlongumine#section=2D-Structure.

[70] Berberine|C20H18NO4+-PubChem. https://pubchem.ncbi.nlm.nih.gov/compound/2353.

[71] Taraxasterol|C30H50O-PubChem. https://pubchem.ncbi.nlm.nih.gov/compound/Taraxasterol.

[72] Vincristine|C46H56N4O10-PubChem. https://pubchem.ncbi.nlm.nih.gov/compound/Vincristine.

[73] Eupatilin|C18H16O7-PubChem. https://pubchem.ncbi.nlm.nih.gov/compound/Eupatilin.

[74] Khandhar A., Patel S.G., Zaveri M., Patel S., Patel A. (2010). Chemistry and Pharmacology of Piper Longum L.. Int. J. Pharm. Sci. Rev. Res..

[75] Yoo E.S., Choo G.S., Kim S.H., Woo J.S., Kim H.J., Park Y.S., Kim B.S.O.O., Kim S.K., Park B.K., Cho S.D. (2019). Antitumor and Apoptosis-inducing Effects of Piperine on Human Melanoma Cells. Anticancer Res..

[76] Sunila E.S., Kuttan G. (2006). Piper longum inhibits VEGF and proinflammatory cytokines and tumor-induced angiogenesis in C57BL/6 mice. Int. Immunopharmacol..

[77] Bezerra D.P., Pessoa C., De Moraes M.O., Saker-Neto N., Silveira E.R., Costa-Lotufo L.V. (2013). Overview of the therapeutic potential of piplartine (piperlongumine). Eur. J. Pharm. Sci..

[78] Meng F.C., Wu Z.F., Yin Z.Q., Lin L.G., Wang R., Zhang Q.W. (2018). Coptidis rhizoma and its main bioactive components: Recent advances in chemical investigation, quality evaluation and pharmacological activity. Chin. Med. (UK).

[79] Kim H.S., Kim M.J., Kim E.J., Yang Y., Lee M.S., Lim J.S. (2012). Berberine-induced AMPK activation inhibits the metastatic potential of melanoma cells via reduction of ERK activity and COX-2 protein expression. Biochem. Pharmacol..

[80] Wirngo F.E., Lambert M.N., Jeppesen P.B. (2016). The physiological effects of dandelion (*Taraxacum officinale*) in type 2 diabetes. Rev. Diabet. Stud..

[81] Ueda J., Tezuka Y., Banskota A.H., Le Tran Q., Tran Q.K., Harimaya Y., Saiki I., Kadota S. (2002). Antiproliferative Activity of Vietnamese Medicinal Plants. Biol. Pharm. Bull..

[82] Groth-Pedersen L., Ostenfeld M.S., Høyer-Hansen M., Nylandsted J., Jäättelä M. (2007). Vincristine induces dramatic lysosomal changes and sensitizes cancer cells to lysosome-destabilizing siramesine. Cancer Res..

[83] Albuquerque K.R.S., Pacheco N.M., Casao T.D.R.L., De Melo F.C.S.A., Novaes R.D., Gonçalves R.V. (2018). Applicability of Plant Extracts in Preclinical Studies of Melanoma: A Systematic Review. Mediat. Inflamm..

[84] Al Shawi A., Rasul A., Khan M., Iqbal F., Tonghui M. (2011). Eupatilin: A flavonoid compound isolated from the artemisia plant, induces apoptosis and G2/M phase cell cycle arrest in human melanoma A375 cells. Afr. J. Pharm. Pharmacol..

[85] Hata K., Ishikawa K., Hori K., Konishi T. (2000). Differentiation-inducing activity of lupeol, a lupane-type triterpene from Chinese dandelion root (Hokouei-kon), on a mouse melanoma cell line. Biol. Pharm. Bull..

[86] Takasaki M., Konoshima T., Tokuda H., Masuda K., Arai Y., Shiojima K., Ageta H. (1999). Anti-carcinogenic activity of Taraxacum plant. II. Biol. Pharm. Bull..

[87] Liu R., Cao Z., Pan Y., Zhang G., Yang P., Guo P., Zhou Q. (2013). Jatrorrhizine hydrochloride inhibits the proliferation and neovascularization of C8161 metastatic melanoma cells. Anticancer. Drugs.

[88] Yan L., Yee J.A., Li D., Mcguire M.H., Graef G.L. (1998). Dietary flaxseed supplementation and experimental metastasis of melanoma cells in mice. Cancer Lett..

[89] Gofita E., Calina D., Blendea A., Brandusa C., Mitrut R. (2017). Curcumin in the Treatment of Melanoma. Trends Toxicol. Relat. Sci..

[90] Chen W., Lu Y., Wu J., Gao M., Wang A., Xu B. (2011). Beta-elemene inhibits melanoma growth and metastasis via suppressing vascular endothelial growth factor-mediated angiogenesis. Cancer Chemother. Pharmacol..

[91] Park S.Y., Jin M.L., Kim Y.H., Kim Y., Lee S.J. (2011). Aromatic-turmerone inhibits α-MSH and IBMX-induced melanogenesis by inactivating CREB and MITF signaling pathways. Arch. Dermatol. Res..

[92] Selimovic D., Badura H.E., El-Khattouti A., Soell M., Porzig B.B.O.W., Spernger A., Ghanjati F., Santourlidis S., Haikel Y., Hassan M. (2013). Vinblastine-induced apoptosis of melanoma cells is mediated by Ras homologous A protein (Rho A) via mitochondrial and non-mitochondrial-dependent mechanisms. Apoptosis.

[93] Faião-Flores F., Suarez J.A.Q., Fruet A.C., Maria-Engler S.S., Pardi P.C., Maria D.A. (2015). Curcumin analog DM-1 in monotherapy or combinatory treatment with dacarbazine as a strategy to inhibit in vivo melanoma progression. PLoS ONE.

[94] Piotrowska A., Wierzbicka J., Rybarczyk A., Tuckey R.C., Slominski A.T., Zmijewski M.A. (2019). Vitamin D and its low calcemic analogs modulate the anticancer properties of cisplatin and dacarbazine in the human melanoma A375 cell line. Int. J. Oncol..

[95] Baharara J., Amini E., Nikdel N., Salek-Abdollahi F. (2016). The cytotoxicity of dacarbazine potentiated by sea cucumber saponin in resistant B16F10 melanoma cells through apoptosis induction. Avicenna J. Med. Biotechnol..

[96] Ellison L.F. (2016). Increasing survival from leukemia among adolescents and adults in Canada: A closer look. Health Rep..

[97] IIsCanada.org Leukemia & Lymphoma Society of Canada. https://www.llscanada.org/disease-information/facts-and-statistics.

[98] Canadian Cancer Statistics Advisory Committee (2019). Canadian Cancer Statistics 2019.

[99] Stöppler M. (2019). Leukemia Treatment, Diagnosis, Causes, Symptoms & Prognosis. https://www.medicinenet.com/leukemia/article.htm.

[100] Titov D., He Q. (2011). Solving A Traditional Chinese Medicine Mystery-03/02/2011. https://www.hopkinsmedicine.org/news/media/releases/solving_a_traditional_chinese_medicine_mystery.

[101] Amato I. An Eye on Cancer, A Smiling Death. https://cen.acs.org/articles/87/i35/Eye-Cancer-Smiling-Death.html..

[102] Siveen K.S., Uddin S., Mohammad R.M. (2017). Targeting acute myeloid leukemia stem cell signaling by natural products. Mol. Cancer.

[103] Bhatia D., Bishayee A. (2019). Epigenetic Dietary Interventions for Cancer Prevention. Epigenetics of Cancer Prevention.

[104] Parthenolide|C15H20O3-PubChem. https://pubchem.ncbi.nlm.nih.gov/compound/108068.

[105] Triptolide Analog|C21H30O5-PubChem. https://pubchem.ncbi.nlm.nih.gov/compound/Triptolide-analog.

[106] Cantharidin|C10H12O4-PubChem. https://pubchem.ncbi.nlm.nih.gov/compound/Cantharidin.

[107] Cyclopamine|C27H41NO2-PubChem. https://pubchem.ncbi.nlm.nih.gov/compound/Cyclopamine.

[108] Rauh R., Kahl S., Boechzelt H., Bauer R., Kaina B., Efferth T. (2007). Molecular biology of cantharidin in cancer cells. Chin. Med..

[109] Lee S.T., Welchrl K.D., Panter K., Gardner D.R., Garrossian M., Chang C.-W.T. (2014). Cyclopamine: From cyclops lambs to cancer treatment. J. Agric. Food Chem..

[110] Mertens-Talcott S.U., Talcott S.T., Percival S.S. (2003). Low concentrations of quercetin and ellagic acid synergistically influence proliferation, cytotoxicity and apoptosis in MOLT-4 human leukemia cells. J. Nutr..

[111] Mertens-Talcott S.U., Percival S.S. (2005). Ellagic acid and quercetin interact synergistically with resveratrol in the induction of apoptosis and cause transient cell cycle arrest in human leukemia cells. Cancer Lett..

[112] Ferlay J., Bray F., Pisani P., Parkin D. (2015). Cancer mortality and mortality worldwide: Sources, methods, and major patterns in GLOBOCAN 2012. Int. J. Cancer.

[113] MacDonald V. (2009). Chemotherapy: Managing side effects and safe handling. Can. Vet. J..

[114] Ovadje P., Ammar S., Guerrero J.A., Arnason J.T., Pandey S. (2016). Dandelion root extract affects colorectal cancer proliferaion and survival through the activation of multiple death signalling pathways. Oncotarget.

[115] Ruvinov I., Nguyen C., Scaria B., Vegh C., Zaitoon O., Baskaran K., Mehaidli A., Nunes M., Pandey S. (2019). Lemongrass extract possesses potent anticancer activity against human colon cancers, inhibits tumorigenesis, enhances efficacy of FOLFOX, and reduces its adverse effects. Integr. Cancer Ther..

[116] Li W., Wen C., Bai H., Wang X., Zhang X., Huang L., Yang X., Iwamoto A., Liu H. (2015). JNK signaling pathway is involved in piperlongumine-mediated apoptosis in human colorectal cancer HCT116 cells. Oncol. Lett..

[117] Ovadje P., Ma D., Tremblay P., Roma A., Steckle M., Guerrero J.A., Arnason J.T., Pandey S. (2014). Evaluation of the efficacy & biochemical mechanism of cell death induction by Piper longum extract selectively in in-vitro and in-vivo models of human cancer cells. PLoS ONE.

[118] Pérez-Sánchez A., Barrajón-Catalán E., Ruiz-Torres V., Agulló-Chazarra L., Herranz-López M., Valdés A., Cifuentes A., Micol V. (2019). Rosemary (*Rosmarinus officinalis*) extract causes ROS-induced necrotic cell death and inhibits tumor growth in vivo. Sci. Rep..

[119] Mosieniak G., Adamowicz M., Alster O., Jaskowiak H., Szczepankiewicz A.A., Wilczynski G.M., Ciechomska I.A., Sikora E. (2012). Curcumin induces permanent growth arrest of human colon cancer cells: Link between senescence and autophagy. Mech. Ageing Dev..

[120] Kim K.C., Lee C. (2010). Curcumin induces downregulation of E2F4 expression and apoptotic cell death in HCT116 human colon cancer cells; involvement of reactive oxygen species. Korean J. Physiol. Pharmacol..

[121] Mishra J., Drummond J., Quazi S.H., Karanki S.S., Shaw J.J., Chen B., Kumar N. (2013). Prospective of colon cancer treatments and scope for combinatorial approach to enhanced cancer cell apoptosis. Crit. Rev. Oncol..

[122] Van Cutsem E., Köhne C.-H., Hitre E., Zaluski J., Chien C.-R.C., Makhson A., D’Haens G., Pintér T., Lim R., Bodoky G. (2009). Cetuximab and chemotherapy as initial treatment for metastatic colorectal cancer. N. Engl. J. Med..

[123] Fulda S., Debatin K.M. (2006). Extrinsic versus intrinsic apoptosis pathways in anticancer chemotherapy. Oncogene.

[124] Goodwin R.A., Asmis T.R. (2009). Overview of systemic therapy for colorectal cancer. Clin. Colon Rectal Surg..

[125] Mann J. (2002). Natural products in cancer chemotherapy: Past, present and future. Nat. Rev. Cancer.

[126] Randhawa H., Kibble K., Zeng H., Moyer M.P., Reindl K.M. (2013). Activation of ERK signaling and induction of colon cancer cell death by piperlongumine. Toxicol. Vitr..

[127] Liu J.M., Pan F., Li L., Liu Q.R., Chen Y., Xiong X.X., Cheng K., Bin Y.S., Shi Z., Yu A.C.-H. (2013). Piperlongumine selectively kills glioblastoma multiforme cells via reactive oxygen species accumulation dependent JNK and p38 activation. Biochem. Biophys. Res. Commun..

[128] Aggarwal B.B., Sung B. (2009). Pharmacological basis for the role of curcumin in chronic diseases: An age-old spice with modern targets. Trends Pharmacol. Sci..

[129] Kisiel W., Barszcz B. (2000). Further sesquiterpenoids and phenolics from Taraxacum officinale. Fitoterapia.

[130] Watson J.L., Hill R., Yaffe P.B., Greenshields A., Walsh M., Lee P.W., Giacomantonio C.A., Hoskin D.W. (2010). Curcumin causes superoxide anion production and p53-independent apoptosis in human colon cancer cells. Cancer Lett..

[131] Arbiser J.L., Klauber N., Rohan R., van Leeuwen R., Huang M.T., Fisher C., Flynn E., Byers H.R. (1998). Curcumin is an in vivo inhibitor of angiogenesis. Mol. Med..

[132] De Ieso M.L., Pei J.V., Nourmohammadi S., Smith E., Chow P.H., Kourghi M., Hardingham J.E., Yool A.J. (2019). Combined pharmacological administration of AQP1 ion channel blocker AqB011 and water channel blocker Bacopaside II amplifies inhibition of colon cancer cell migration. Sci. Rep..

[133] Manach C., Scalbert A., Morand C., Rémésy C., Jiménez L. (2004). Polyphenols: Food sources and bioavailability. Am. J. Clin. Nutr..

[134] Gonzalez-Castejon M., Visioli F., Rodriguez-Casado A. (2012). Diverse biological activities of dandelion. Nutr. Rev..

[135] Xue Y., Zhang S., Du M., Zhu M.J. (2017). Dandelion extract suppresses reactive oxidative species and inflammasome in intestinal epithelial cells. J. Funct. Foods.

[136] Chen H.J., Inbaraj B.S., Chen B.H. (2012). Determination of phenolic acids and flavonoids in Taraxacum formosanum Kitam by liquid chromatography-tandem mass spectrometry coupled with a post-column derivatization technique. Int. J. Mol. Sci..

[137] Hu C., Kitts D.D. (2005). Dandelion (*Taraxacum officinale*) flower extract suppresses both reactive oxygen species and nitric oxide and prevents lipid oxidation in vitro. Phytomedicine.

[138] Chicoric Acid|C22H18O12-PubChem. https://pubchem.ncbi.nlm.nih.gov/compound/Chicoric-acid.

[139] Chlorogenic Acid|C16H18O9-PubChem. https://pubchem.ncbi.nlm.nih.gov/compound/Chlorogenic-acid.

[140] Carnosic Acid|C20H28O4-PubChem. https://pubchem.ncbi.nlm.nih.gov/compound/Carnosic-acid.

[141] Carnosol|C20H26O4-PubChem. https://pubchem.ncbi.nlm.nih.gov/compound/Carnosol.

[142] Halabi M.F., Sheikh B.Y. (2014). Anti-proliferative effect and phytochemical analysis of Cymbopogon citratus extract. BioMed Res. Int..

[143] Olorunnisola K.S., Hammed A.M., Asiyanbi-Hammed T., Simsek S. (2014). Biological properties of lemongrass: An overview. Int. Food Res. J..

[144] Hu R., Kim B.R., Chen C., Hebbar V., Kong A.N. (2003). The roles of JNK and apoptotic signaling pathways in PEITC-mediated responses in human HT-29 colon adenocarcinoma cells. Carcinogenesis.

[145] Xu C., Shen G., Yuan X., Kim J.H., Gopalkrishnan A., Keum Y.S., Nair S., Kong A.T. (2006). ERK and JNK signaling pathways are involved in the regulation of activator protein 1 and cell death elicited by three isothiocyanates in human prostate cancer PC-3 cells. Carcinogenesis.

[146] Visanji J.M., Thompson D.G., Padfield P.J. (2006). Induction of G2/M phase cell cycle arrest by carnosol and carnosic acid is associated with alteration of cyclin A and cyclin B1 levels. Cancer Lett..

[147] Johnson J.J. (2011). Carnosol: A promising anti-cancer and anti- inflammatory agent. Cancer Lett..

[148] Valdés A., García-Cañas V., Simó C., Ibáñez C., Micol V., Ferragut J.A., Cifuentes A. (2014). Comprehensive foodomics study on the mechanisms operating at various molecular levels in cancer cells in response to individual rosemary polyphenols. Anal. Chem..

[149] Kunnumakkara A.B., Bordoloi D., Padmavathi G., Monisha J., Roy N.K., Prasad S., Aggarwal B.B. (2017). Curcumin, the golden nutraceutical: Multitargeting for multiple chronic diseases. Br. J. Pharmacol..

[150] Su C.C., Lin J.G., Li T.M., Chung J.G., Yang J.S., Ip S.W., Lin W.C., Chen G.W. (2006). Curcumin-induced apoptosis of human colon cancer colo 205 cells through the production of ROS, Ca2+ and the activation of caspase-3. Anticancer Res..

[151] Collett G.P., Campbell F.C. (2006). Overexpression of p65/RelA potentiates curcumin-induced apoptosis in HCT116 human colon cancer cells. Carcinogenesis.

[152] Gandhy S.U., Kim K., Larsen L., Rosengren R.J., Safe S. (2012). Curcumin and synthetic analogs induce reactive oxygen species and decreases specificity protein (Sp.) transcription factors by targeting microRNAs. BMC Cancer.

[153] Park C.M., Jin K.S., Lee Y.W., Song Y.S. (2011). Luteolin and chicoric acid synergistically inhibited inflammatory responses via inactivation of PI3K-Akt pathway and impairment of NF-κB translocation in LPS stimulated RAW 264.7 cells. Eur. J. Pharmacol..

[154] Philion C., Ma D., Ruvinov I., Mansour F., Pignanelli C., Noel M., Saleem A., Arnason J., Rodrigues M., Singh I. (2017). *Cymbopogon citratus* and *Camellia sinensis* extracts selectively induce apoptosis in cancer cells and reduce growth of lymphoma xenografts in vivo. Oncotarget.

[155] Du B., Jiang L., Xia Q., Zhong L. (2006). Synergistic inhibitory effects of curcumin and 5-fluorouracil on the growth of the human colon cancer cell line HT-29. Chemotherapy.

[156] Nautiyal J., Kanwar S.S., Yu Y., Majumdar A.P. (2011). Combination of dasatinib and curcumin eliminates chemo-resistant colon cancer cells. J. Mol. Signal..

[157] Han W., Xie B., Li Y., Shi L., Wan J., Chen X., Wang H. (2019). Orally deliverable nanotherapeutics for the synergistic treatment of colitis-associated colorectal cancer. Theranostics.

[158] Pancreatic Cancer Statistics-Canadian Cancer Society. https://www.cancer.ca/en/cancer-information/cancer-type/pancreatic/statistics/?region=on.

[159] Survival Rates for Pancreatic Cancer. American Cancer Society. Published 2020. https://www.cancer.org/cancer/pancreatic-cancer/detection-diagnosis-staging/survival-rates.html#references.

[160] Lung Cancer Statistics-Canadian Cancer Society. https://www.cancer.ca/en/cancer-information/cancer-type/lung/statistics/?region=pe.

[161] Kamisawa T., Wood L.D., Itoi T., Takaori K. (2016). Pancreatic cancer. Lancet.

[162] Kunk P.R., Bauer T.W., Slingluff C.L., Rahma O.E. (2016). From bench to bedside a comprehensive review of pancreatic cancer immunotherapy. J. Immunother. Cancer.

[163] Cooper S., Spiro S.G. (2006). Small cell lung cancer: Treatment review. Respirology.

[164] Hirsch F.R., Scagliotti G.V., Mulshine J.L., Kwon R., Curran W.J., Wu Y.-L., Paz-Ares L. (2017). Lung cancer: Current therapies and new targeted treatments. Lancet.

[165] Zappa C., Mousa S.A. (2016). Non-small cell lung cancer: Current treatment and future advances. Transl. Lung Cancer Res..

[166] Kummalue T. (2005). Molecular Mechanism of Herbs in Human Lung Cancer Cells. https://www.researchgate.net/publication/51373862.

[167] Li L., Leung P.S. (2014). Use of herbal medicines and natural products: An alternative approach to overcoming the apoptotic resistance of pancreatic cancer. Int. J. Biochem. Cell Biol..

[168] Zhang J.-H., Lai F.-J., Chen H., Luo J., Zhang R.-Y., Bu H.-Q., Wang Z.-H., Lin H.-H., Lin S.-Z. (2013). Involvement of the phosphoinositide 3-kinase/Akt pathway in apoptosis induced by capsaicin in the human pancreatic cancer cell line PANC-1. Oncol. Lett..

[169] Wang Q., Zheng X.-L., Yang L., Shi F., Gao L., Zhong Y.-J., Sun H., He F., Lin Y., Wang X. (2010). Reactive oxygen species-mediated apoptosis contributes to chemosensitization effect of saikosaponins on cisplatin-induced cytotoxicity in cancer cells. J. Exp. Clin. Cancer Res..

[170] Hsu Y.-L., Kuo P.-L., Chiang L.-C., Lin C.-C. (2004). Isoliquiritigenin Inhibits the Proliferation and Induces the Apoptosis of Human Non-Small Cell Lung Cancer a549 Cells. Clin. Exp. Pharmacol. Physiol..

[171] Capsaicin|C18H27NO3-PubChem. https://pubchem.ncbi.nlm.nih.gov/compound/Capsaicin.

[172] Genistein|C15H10O5-PubChem. https://pubchem.ncbi.nlm.nih.gov/compound/Genistein.

[173] Ginsenosides|C30H52O2-PubChem. https://pubchem.ncbi.nlm.nih.gov/compound/Ginsenosides.

[174] Saikosaponin D|C42H68O13-PubChem. https://pubchem.ncbi.nlm.nih.gov/compound/Saikosaponin-D.

[175] Isoliquiritigenin|C15H12O4-PubChem. https://pubchem.ncbi.nlm.nih.gov/compound/Isoliquiritigenin.

[176] Meng F.-C., Zhou Y.-Q., Ren D., Wang R., Wang C., Lin L.-G., Zhang X.-Q., Ye W.-C., Zhang Q.-W. (2018). Turmeric: A Review of Its Chemical Composition, Quality Control, Bioactivity, and Pharmaceutical Application. Natural and Artificial Flavoring Agents and Food Dyes.

[177] Li S., Yuan W., Deng G., Wang P., Yang P., Aggarwal B. Chemical Composition and Product Quality Control of Turmeric (*Curcuma longa* L.). Fac Publ. Published Online 1 January 2011. https://scholarworks.sfasu.edu/agriculture_facultypubs/1.

[178] Hwang S.-H., Lee B.-H., Kim H.-J., Cho H.-J., Shin H.-C., Im K.-S., Choi S.-H., Shin T.-J., Lee S.-M., Nam S.W. (2012). Suppression of metastasis of intravenously-inoculated B16/F10 melanoma cells by the novel ginseng-derived ingredient, gintonin: Involvement of autotaxin inhibition. Int. J. Oncol..

[179] Torre L.A., Bray F., Siegel R.L., Ferlay J., Lortet-Tieulent J., Jemal A. (2015). Global cancer statistics, 2012: Global Cancer Statistics, 2012. CA Cancer J. Clin..

[180] Siegel R.L., Miller K.D., Jemal A. (2019). Cancer statistics, 2019. CA Cancer J. Clin..

[181] Thompson I., Thrasher J.B., Aus G., Burnett A.L., Canby-Hagino E.D., Cookson M.S., D’Amico A.V., Dmochowski R.R., Eton D.T., Forman J.D. (2007). Guideline for the Management of Clinically Localized Prostate Cancer: 2007 Update. J. Urol..

[182] Chen F., Zhao X. (2013). Prostate Cancer: Current Treatment and Prevention Strategies. Iran. Red Crescent Med. J..

[183] Procházková D., Boušová I., Wilhelmová N. (2011). Antioxidant and Prooxidant Properties of Flavonoids. Fitoterapia.

[184] Lafay S., Gueux E., Rayssiguier Y., Mazur A., Rémésy C., Scalbert A. (2005). Caffeic Acid Inhibits Oxidative Stress and Reduces Hypercholesterolemia Induced by Iron Overload in Rats. Int. J. Vitam. Nutr. Res..

[185] Caffeic Acid|C9H8O4-PubChem. https://pubchem.ncbi.nlm.nih.gov/compound/Caffeic-acid#section=2D-Structure.

[186] Elemicin|C12H16O3-PubChem. https://pubchem.ncbi.nlm.nih.gov/compound/Elemicin#section=2D-Structure.

[187] Gallic Acid|C7H6O5-PubChem. https://pubchem.ncbi.nlm.nih.gov/compound/Gallic-acid#section=2D-Structure.

[188] You Q., Ni H., Sharp J.L., Wang X., You Y., Zhang C. (2012). High-Performance Liquid Chromatography-Mass Spectrometry and Evaporative Light-Scattering Detector to Compare Phenolic Profiles of Muscadine Grapes. J. Chromatogr. A.

[189] Hudson T.S., Hartle D.K., Hursting S.D., Nunez N.P., Wang T.T., Young H.A., Arany P., Green J.E. (2007). Inhibition of Prostate Cancer Growth by Muscadine Grape Skin Extract and Resveratrol through Distinct Mechanisms. Cancer Res..

[190] Lewandowska H., Kalinowska M., Lewandowski W., Stępkowski T.M., Brzóska K. (2016). The Role of Natural Polyphenols in Cell Signaling and Cytoprotection against Cancer Development. J. Nutr. Biochem..

[191] Erdogan S., Doganlar O., Doğanlar Z.B., Serttas R., Turkekul K., Dibirdik I., Bilir A. (2016). The Flavonoid Apigenin Reduces Prostate Cancer CD44+ Stem Cell Survival and Migration through PI3K/Akt/NF-ΚB Signaling. Life Sci..

[192] Zheng G.Q., Kenney P.M., Lam L.K.T. (1993). Potential Anticarcinogenic Natural Products Isolated from Lemongrass Oil and Galanga Root Oil. J. Agric. Food Chem..

[193] Sharmila G., Bhat F., Arunkumar R., Elumalai P., Singh P.R., Senthilkumar K., Arunakaran J. (2014). Chemopreventive Effect of Quercetin, a Natural Dietary Flavonoid on Prostate Cancer in Invivo Model. Clin. Nutr..

[194] Ignacio D.N., Mason K.D., Hackett-Morton E.C., Albanese C., Ringer L., Wagner W.D., Wang P.C., Carducci M.A., Kachhap S.K., Paller C.J. (2019). Muscadine Grape Skin Extract Inhibits Prostate Cancer Cells by Inducing Cell-Cycle Arrest, and Decreasing Migration through Heat Shock Protein 40. Heliyon.

[195] Madonna G., Ullman C.D., Gentilcore G., Palmieri G., Ascierto P.A. (2012). NF-κB as potential target in the treatment of melanoma. J. Transl. Med..

[196] Herranz-López M., Losada-Echeberría M., Barrajón-Catalán E. (2018). The Multitarget Activity of Natural Extracts on Cancer: Synergy and Xenohormesis. Medicines.

[197] Afshari K., Haddadi N.S., Haj-Mirzaian A., Farzaei M.H., Rohani M.M., Akramian F., Naseri R., Sureda A., Ghanaatian N., Abdolghaffari A.H. (2019). Natural flavonoids for the prevention of colon cancer: A comprehensive review of preclinical and clinical studies. J. Cell. Physiol..

[198] Saif M.W., Lansigan F., Ruta S., Lamb L., Mezes M., Elligers K., Grant N., Jiang Z.L., Liu S.H., Cheng Y.C. (2010). Phase I study of the botanical formulation PHY906 with capecitabine in advanced pancreatic and other gastrointestinal malignancies. Phytomedicine.

[199] Saif M.W., Li J., Lamb L., Kaley K., Elligers K., Jiang Z., Bussom S., Liu S.H., Cheng Y.C. (2014). First-in-human phase II trial of the botanical formulation PHY906 with capecitabine as second-line therapy in patients with advanced pancreatic cancer. Cancer Chemother. Pharmacol..

[200] Yue Q., Gao G., Zou G., Yu H., Zheng X. (2017). Natural Products as Adjunctive Treatment for Pancreatic Cancer: Recent Trends and Advancements. BioMed Res. Int..

